# Xylem Pit Anatomy and Minimum Leaf Conductance Drive Drought Mortality in *Pinus pinaster*

**DOI:** 10.1111/pce.70211

**Published:** 2025-09-30

**Authors:** J. Julio Camarero, Michele Colangelo, Cristina Valeriano, Antonio Gazol, Ester González de Andrés, David Alonso‐Forn, Jordi Voltas, José M. Torres‐Ruiz, Sylvain Delzon, Eric Badel, Eustaquio Gil‐Pelegrín

**Affiliations:** ^1^ Instituto Pirenaico de Ecología (IPE‐CSIC) Zaragoza Spain; ^2^ Dipartimento di Scienze Agrarie, Forestali, Alimentari e Ambientali Università della Basilicata Potenza Italy; ^3^ Research Group on Plant Biology under Mediterranean conditions, Departament de Biologia Universitat de les Illes Balears/Institute of Agro‐Environmental Research and Water Economy –INAGEA Palma Spain; ^4^ Department of Agricultural and Forest Sciences and Engineering University of Lleida Lleida Spain; ^5^ Joint Research Unit CTFC‐AGROTECNIO‐CERCA Lleida Spain; ^6^ Instituto de Recursos Naturales y Agrobiología de Sevilla (IRNAS), Consejo Superior de Investigaciones Científicas (CSIC), Avda Reina Mercedes Sevilla Spain; ^7^ INRAE, UMR BIOGECO University of Bordeaux Pessac France; ^8^ INRAE, PIAF Université Clermont Auvergne Clermont‐Ferrand France; ^9^ Estación Experimental de Aula Dei, Consejo Superior de Investigaciones Científicas (EEAD‐CSIC) Zaragoza Spain

**Keywords:** drought, forest die‐off, minimum leaf conductance, pit anatomy, tree death

## Abstract

Drought‐triggered forest die‐off events are commonly attributed to hydraulic failure, carbon starvation, or a combination of the two. Nevertheless, the anatomical and physiological traits that make trees vulnerable to drought in the field are often unknown, hindering predictive efforts. To identify these traits, we compared coexisting declining (D, heavily defoliated) and non‐declining (ND, lightly defoliated) trees. We studied a recent die‐off event affecting maritime pine (*Pinus pinaster*) in north‐eastern Spain that started after the severe 2017 drought. We compared the depth of soil water uptake, estimated using δ^18^O and δ^2^H in soil and xylem water samples, as well as field measurements. We also measured anatomical and physiological wood and leaf variables, paying particular attention to pit anatomy and minimum leaf conductance (*g*_*min*_). The D trees were smaller in terms of diameter and height, and exhibited lower growth rates. They also formed tracheids with smaller lumen diameters and thinner cell walls than the ND trees. The measured soil depth was greater for ND than for D trees. Isotope data also indicated that ND trees used water from deeper soil layers than D trees during the late summer period of peak drought severity. No differences in the sapwood concentrations of non‐structural carbohydrates were found between the two tree types. The D trees had lower midday water potentials than ND trees, and the pressure inducing 50% loss of hydraulic conductance (P_50_) and *g*_*min*_ were higher in D trees. The D trees also exhibited lower torus overlap, margo flexibility and valve effect than ND trees. However, these differences in pit anatomy were observed in the 2010s when ND trees exhibited higher δ^13^C‐derived intrinsic water‐use efficiency. A combination of traits, such as a large pit aperture and a high *g*_*min*_ makes trees vulnerable to drought stress.

## Summary statement

We investigated why some *Pinus pinaster* trees die during droughts while others survive. Declining trees have lower torus overlap (a trait affecting water transport safety) and higher minimum leaf conductance (leading to greater water loss), which together make them more vulnerable to drought stress.

## 1 Introduction

Events of forest die‐off and elevated mortality rates triggered by severe and hotter droughts have been reported for all forested biomes (Allen et al. 2010, 2015; Brodribb et al. 2020; Hammond et al. 2022). Such impacts could be amplified by climate warming and aridification, increasing the risk of tree mortality (Williams et al. 2013; McDowell et al. 2022). Two major mechanisms related to hydraulic failure, that is, a progressive reduction of the xylem hydraulic functioning, and carbon starvation, that is, an impairment of sources or fluxes to supply sufficient carbon to metabolism, have been proposed to explain tree death due to drought stress (McDowell et al. 2008). However, many studies have shown that under fast dehydration and severe drought conditions, hydraulic failure is the mechanism which plays a major role in explaining drought‐induced tree mortality (Rowland et al. 2015; Anderegg et al. 2016; Adams et al. 2017; Mantova et al. 2022). For instance, Arend et al. (2021) found that dying Norway spruce (*Picea abies* L.) trees during a record drought showed hydraulic collapse. They ruled out carbon starvation as the cause of tree death, as both dying and surviving trees showed similar non‐structural carbohydrate (NSCs) concentrations.

Under this scenario, it is crucial to understand the anatomical and physiological traits determining hydraulic dysfunction and its impacts to improve forecasts of tree drought mortality (Trugman et al. 2001). A widely used measure of the xylem resistance to cavitation is the tension inducing 50% loss of hydraulic conductance (P_50_), which is generally used as the lethal percent loss of hydraulic conductance (PLC) for conifers (Brodribb and Cochard 2009), despite higher lethal PLCs having been reported for some species (Hammond et al. 2019; Mantova et al. 2021). Therefore, determining the variation in tree water status (through water potential measurements) and P_50_ is needed for the hydraulic characterisation of declining trees and can improve our predictive power for quantifying the impacts of drought on forests (Torres‐Ruiz et al. 2024). However, these measures require linkages with other critical processes and characteristics related to soil water availability, carbon sinks and sources (meristems), including soil water uptake, gas‐water exchange, wood anatomy and tree growth (Mantova et al. 2022). In addition, coexisting trees could be differently predisposed to xylem embolism and meristem damage as a function of environmental factors (e.g., soil depth, percent rock content). Therefore, the access to soil water sources during the dry season should be measured using water isotope composition (δ^18^O and δ^2^H) to assess such predisposition to drought damage (Ripullone et al. 2020).

Considering xylem traits in conifers, it has been found that trees prone to growth decline and death due to drought stress show lower growth rates and form tracheids with smaller lumen area (Camarero et al. 2015; Pellizzari et al. 2016). In the study by Pellizzari et al. (2016), declining Scots pine (*Pinus sylvestris* L.) trees in a xeric site also showed higher δ^13^C‐derived intrinsic water‐use efficiency (iWUE, or the ratio of photosynthesis to stomatal conductance), suggesting a water‐saving strategy. However, the opposite was observed in a mesic silver fir (*Abies alba* Mill.) site, where declining trees exhibited a water‐spending strategy. In addition, cavitation resistance is correlated to the ‘thickness to span’ ratio of tracheids (Hacke et al. 2001) and the torus overlap (ratio of torus to pit aperture diameter) (Delzon et al. 2010). Thus, conifer species with higher cavitation resistance show large torus overlap, mainly because of decreased aperture diameter and valve effect, that is, the capacity of the torus to seal the aperture during pit aspiration (Bouche et al. 2014). Besides, slow‐growing trees also show large torus overlap (Roskilly et al. 2019). Nevertheless, xylem pit anatomy has not been analysed as a factor contributing to drought‐related tree death despite its primary role in determining cavitation resistance and hydraulic failure.

Compound climate events characterised by heat and drought stresses may amplify the risk of hydraulic failure and cause tree death (Gazol and Camarero 2022). Physiologically, this could be explained by a nonlinear increase of residual leaf transpiration, linked to cuticle phase transition temperatures, under elevated leaf‐to‐air vapour pressure deficit (VPD) and hot conditions (Martin‐StPaul et al. 2017; Cochard 2021). Such excessive residual water loss would be caused by high cuticular permeability and trigger xylem embolism and hydraulic failure, leading to tree death (Blackman et al. 2023; Petek‐Petrik et al. 2023). This highlights the role played by the minimum leaf conductance (*g*_*min*_), that is, the lowest conductance a leaf can reach when stomata are closed due to desiccation stress (Körner 1995), to explain tree resistance to heat and drought stress (Duursma et al. 2019). The *g*_*min*_ is a good proxy for leaf cuticular water permeability, and it is known to be very influenced by leaf temperature (Burghardt 2003). Some studies have shown a sharp increase in *g*_*min*_ in response to high temperatures during hot droughts (Riederer and Müller 2006). However, the role played by *g*_*min*_ as related to leaf water loss in processes of drought tree mortality has not been properly assessed by comparing declining and non‐declining individuals.

Here, we investigate a battery of soil (depth of water sources), leaf and wood variables to decipher the main mechanisms involved in a drought‐triggered die‐off affecting a Mediterranean *Pinus pinaster* Ait. forest. We compared coexisting trees showing different canopy defoliation, a proxy of tree vigour, in response to drought. We focused on leaf (*g*_*min*_) and wood (torus overlap) traits, which could explain the divergent responses of the two vigour classes. We hypothesise that declining trees, showing higher defoliation, will: (i) have reduced access to deep water sources during the late summer when the water deficit is at its peak; (ii) experience slower growth and form tracheids with a narrower lumen; (iii) exhibit higher *g*_*min*_; and (iv) demonstrate greater vulnerability to xylem cavitation, due to smaller torus overlap and/or larger pit apertures.

## 2 Materials and Methods

### 2.1 Study Site and Climate Data

We selected a site showing ongoing die‐off and located near Miedes de Aragón, north eastern Spain (1.4337° W, 41.2686° N, 961 m a.s.l., exposition was 240°, mean slope 5%). The site is a natural *P. pinaster* stand with abundant canopy dieback and patches of high mortality (11%−25% of trees; see Valeriano, Gazol, Colangelo, González de Andrés, et al. 2021; Camarero et al. 2025). The mean density and basal area are 695 stems ha^−1^ and 30 m^2^ ha^−1^, respectively. Tree‐to‐tree competition is low, and the stand has been subjected to light thinning in the past. The second most abundant species forming the mid‐story (height 2−3 m) was the Holm oak (*Quercus ilex* L.), which did not show die‐off. We measured some variables (PLC, water isotopes) in *Q. ilex* to use it as a reference drought‐tolerant species with respect to *P. pinaster*. The understory was dominated by *Juniperus oxycedrus* L., *Arctostaphylos uva‐ursi* (L.) Spreng. and *Lavandula stoechas* Lam. The main geological substrates are quartzites. Soils are acidic (pH = 5.6) and rocky with sandy‐loamy or clay texture (Gazol et al. 2024), and are relatively shallow (20–50 cm). The amount of organic nitrogen in the soil is low (0.12%).

Forest managers first noticed signs of die‐off in 2017, following a severe drought (Camarero et al. 2025). However, no pathogens or insect defoliators were identified. Canopy dieback was patchy, being more intense on south‐facing slopes with rocky, thin soils, that is, areas that receive more sunlight and are warmer and drier (Moreno‐Fernández et al. 2021, 2022). Some sites exhibited elevated mortality rates affecting up to 35% of mature pines (Valeriano, Gazol, Colangelo, González de Andrés, et al. 2021; Valeriano, Gazol, Colangelo, et al. 2021).

To characterise climate conditions and trends, we obtained monthly climate variables (mean maximum and minimum temperatures, total precipitation) from the Daroca meteorological station (41.11° N, 1.41° W, 779 m; period 1920−2018), located at ca. 17 km from the study site. We converted these data into seasonal climate variables and also calculated annual climate water balances as the differences between precipitation and potential evapotranspiration (PET). The PET was calculated using the FAO‐56 Penman–Monteith equation. We also considered daily climate data during the monitoring period (2016−2020).

The mean annual temperature in Daroca is 12.0°C, and the total annual precipitation is 423 mm, with a mean annual water balance of −281 mm (Figure S1). The seasonal temperatures have shown long‐term positive trends in the study area, particularly in summer (Figure S2). According to annual water balance data, the 2017 drought was the most extreme since 1920 (Figure S3). During the main monitoring period (2016−2020), conditions were exceptionally warm and dry during 2017 (Figure S4). According to drought indices, the 2017 drought severity peaked from September to December (Camarero et al. 2025).

### 2.2 Field Sampling

At the study site, we selected and tagged 30 dominant trees with contrasting levels of canopy defoliation. We mapped them and measured their diameter at breast height (1.3 m) and height using tapes and a laser rangefinder (Nikon Forestry Pro II). We also estimated the depth of the soil at each tree's location by driving an iron bar into the ground twice under the canopy of each tree until it touched the bedrock.

We monitored their defoliation from 2020 to 2024. Defoliation was visually assessed by three observers in late summer to avoid bias, and then the mean value for each tree was calculated. We used crown defoliation as a proxy of tree vigour and measured it in classes of 5% following the European ICP‐Forest network methodology (Eichhorn et al. 2016). Then, declining (D, *n* = 15) and non‐declining (ND, *n* = 15) trees were classified as those showing crown defoliation levels higher than 60% and lower than 40%, respectively (Camarero et al. 2025).

### 2.3 Soil and Xylem Water Sampling and Extraction, Groundwater Collection and Isotopic Analysis

The soil and stem xylem sampling were carried out in spring (23 June) and late summer (8 September) during 2020. Information on the use of soil water sources for each *P. pinaster* tree class (D, ND) and also for five coexisting *Q. ilex* trees was obtained using measurements of water isotope ratios (oxygen and hydrogen isotope composition, δ^18^O and δ^2^H) for different soil depths as well as for xylem water (Craig 1961; Martín‐Gómez et al. 2015; Grossiord et al. 2017).

Xylem samples were obtained between 11:00 and 13:00 solar time. Two branches were sampled from the upper canopy of each tree on its north‐ and south‐facing sides using telescopic loppers. The sampled branches were approximately 1.5–2.0 cm thick, and shoot segments (5–7 cm long) were cut. The bark was peeled off, and the segments were placed immediately into glass vials and frozen in dry ice to prevent evaporation. Soil samples were collected on the same days at two depths (0–15 and 15–30 cm) until bedrock was reached, using a straight tube probe that was carefully cleaned between consecutive samplings. Between 07:00 and 09:00 solar time, samples were taken from soil pits located between pairs of sampled D and ND trees. The extracted soil was rapidly placed into glass vials and frozen in dry ice. All samples were kept frozen until processing and analysis.

Xylem and soil water were extracted by cryogenic vacuum distillation (Martín‐Gómez et al. 2015). Sample tubes were placed in a heated silicone oil bath (110°C–120°C) and connected with Ultra‐Torr unions (Swagelok Co., Solon, OH, USA) to a vacuum system for a constant vacuum pressure, including *U*‐shaped water traps in series that were refrigerated with liquid N_2_. The extraction time was 90 min for xylem and 120 min for soil samples, which is enough to extract all available water from soil and xylem samples in similar Mediterranean forests (Martín‐Gómez, Serrano and Ferrio 2017). The extracted water was transferred into cap‐crimp 2‐mL vials and stored at 4°C until analysis. Gravimetric soil water content was assessed for each sample using the sample weight before and after water extraction. A subset of representative samples, including both xylem and soil samples, was checked for complete water extraction by oven drying them at 105°C for 36 h and reweighing them. Samples did not show a significant weight loss when placed in the drying oven.

The oxygen (δ^18^O) and hydrogen (δ^2^H) isotopic compositions of the water were determined using isotope‐ratio infra‐red spectroscopy (IRIS) with a Picarro L2120‐i coupled to a high‐precision A0211 vaporiser (Picarro Inc., Sunnyvale, CA, USA). Based on the repeated analysis of four reference water samples, the estimated precision was 0.09‰ for δ^18^O and 0.38‰ for δ^2^H. The presence of residual organic contaminants in the distilled water was tested using Picarro's ChemCorrect post‐processing software, with corrections made where necessary following Martín‐Gómez et al. (2015). The global meteoric water line was described as δ^2^H = 8.06 + 7.77 × δ^18^O (*R* ^2^ = 0.99). For the groundwater samples, the average was taken from water collected from a well (three samples) and the Perejiles river (two samples), both of which were located within a 5 km radius of the sampled stand. These water samples were obtained on the same sampling dates.

### 2.4 Leaf Traits and Water Potential

The leaf traits and *g*_*min*_ were measured in the monitored D and ND trees. Shortly, we collected two sun‐exposed branches from the upper canopy of each tree and selected five 1‐year‐old needles per branch (*n* = 20 needles per tree, that is, 10 fascicles with 2 needles per fascicle). Needles were fully hydrated in a humid chamber overnight before the measurements. Then, the water‐saturated mass was determined using an analytical balance (MC‐1 AC210S, Sartorius, Germany; precision 0.1 mg). The leaf area was measured in high‐resolution images of the needles, obtained in a flatbed scanner (Epson, Expression 10000XL, Japan), using the ImageJ image analysis software (NIH, Bethesda, USA). The needle dry mass was obtained after oven drying the samples at 90°C for 24 h. This allowed calculating the leaf mass per area as the ratio between leaf dry mass and area (g m^−2^).

To measure *g*_*min*_ we selected 10 needles per tree and followed the methodology proposed by Bueno et al. (2020, 2022). Briefly, the *g*_*min*_ was determined by measuring consecutive weight loss of desiccating needles in darkness and at close to 0% relative humidity. The basal part of water‐saturated needles was sealed with high‐melting paraffin wax (Paraplast Plus, Leica, USA) and placed in a closed container with a temperature ca. 25°C. Silica gel (Envirogel, London, UK) was used to control the moisture, obtaining a relative humidity close to 0%. The weight of desiccating needles was determined as a function of desiccation time using an analytical balance.

The water potential was measured at predawn and midday in 1‐year‐old shoots sampled from the upper canopy of each tree. This was done on three dates during 2020, reflecting spring (22 June), mid‐ (28 July) and late‐summer (8 September) conditions. The water potential was measured using a Scholander pressure chamber following recommendations by Rodriguez‐Dominguez et al. (2022).

### 2.5 Vulnerability to Xylem Embolism Formation and Native Embolism

The resistance to xylem embolism is usually assessed by building vulnerability curves, which represent the variation in specific hydraulic conductance (*Ks*, kg s^−1^ MPa^−1^ m^−1^) as a function of xylem water potential (Sperry et al. 1988). In other words, these curves describe how the PLC increases when xylem water potential decreases. Vulnerability curves were built using the flow‐centrifuge (Cavitron) method and following Cochard et al. (2005) and Bouche et al. (2016) methods. This technique is based on the spinning of xylem samples in a custom‐built rotor that induces a wide range of pressures at the centre of a sample. Briefly, one 50–75‐cm‐long branch was sampled from the upper canopy of D and ND trees in June 2020 before sunrise (between 06:00 h and 07:00 h solar time). The excised branches were wrapped with wet paper towels and parafilm, placed in large black plastic bags to prevent water loss, and then transported to the laboratory within 1 h.

In the laboratory, before measurement, all branches were cut under water to a standard length of 27 cm, bark was removed with a razor blade, and then kept in a dark room to release xylem tension (Torres‐Ruiz et al. 2015). Samples were infiltrated with a reference ionic solution of 10 mM 25 KCl and 1 mm CaCl_2_ in deionised ultrapure water. Centrifugal force was used to generate negative pressure in the xylem and to induce cavitation. Initially, the maximum conductance of the stem (*K*_*max*_) was calculated under very low xylem pressures (close to −0.5 MPa). Then, the rotation speed of the centrifuge was gradually increased by 0.5‐MPa steps to a more negative xylem pressure. The pressure gradient was from −1 to −5 MPa. The PLC was determined at each pressure step using the equation:

(1) $$\text{PLC}\;=100\times \left(1–Ks/K_{\max}\right)$$

The relationship between xylem pressure and PLC represents the vulnerability curve of the sample, which was adjusted using sigmoid functions (Pammenter and Vander Willigen 1998). This allowed calculating the xylem pressures inducing 50% loss of conductance (P_50_) in ND and D trees.

Native xylem embolism was assessed by direct observation using X‐ray microtomography (Micro‐CT, Nanotom 180 XS; GE) at the PIAF laboratory (INRAE, France) (see Torres‐Ruiz et al. 2015, 2016 for details of the micro‐CT technique). Briefly, between 7 and 10 trees were sampled for each tree type (D and ND individuals) on three different dates during the summer of 2020: 22 June (7 trees per type), 27 July (9 trees per type) and 8 September (10 trees per type). From each tree, a single ∼1‐cm diameter side branch ∼50 cm in length was cut in the air in the early morning, immediately wrapped in a moist paper towel, placed inside humidified plastic bags, and shipped to the PIAF laboratory. In the laboratory, individual branches were immersed in water and were gradually cut back carefully from each end to a length of ∼20 cm. Samples were then coated in paraffin wax before being stored in a cold room and subsequently scanned. Once the micro‐CT 3D volume was obtained, we virtually extracted the 2D image of the central cross‐section. The total native embolism was determined by evaluating the ratio of embolized tracheids to functional ones, thanks to the Image J software ver. 1.54i (Schneider et al. 2012) and home‐made macros.

### 2.6 Tree‐Ring Width Data

To assess growth changes, two 5‐mm cores per tree were taken at 1.3 m using a Pressler increment borer from D (*n* = 30) and ND (*n* = 30) trees. Wood samples for tree‐ring measurements were air‐dried, glued onto wooden mounts, and surfaced with progressively finer grades of sandpaper until tree rings were clearly distinguishable (Fritts 2012). Then, they were scanned at 2400 dpi resolution using a flatbed scanner (Epson Expression 10000XL) and visually cross‐dated. Ring widths were measured with a 0.001 mm resolution using the CDendro‐CooRecorder software (Maxwell and Larsson 2021). The visual cross‐dating was checked using the COFECHA software, which was used to calculate 20‐year moving correlations between individual series and the mean series of each species (Holmes 1983).

Radial growth variability and trends were quantified as basal area increment (BAI) from the bark towards the pith (outside in), assuming concentric radial growth. The BAI was calculated using the following formula:

(2) $$\text{BAI}\;=\pi (r_{t+1}^{2}-r^{2})$$

where *r*_*t*+1_ and *r* are the cumulative radial increment for years *t* + 1 and *t*, respectively. Lastly, tree age at 1.3 m was estimated by counting the number of rings along the oldest core of each tree whenever it reached the pith or presented curved, innermost rings.

These procedures were done using the *dplR* package (Bunn 2008, 2010; Bunn et al. 2024) in the R statistical software (R Core Team 2024).

### 2.7 Wood Anatomy

In addition to the 5‐mm cores, 10‐mm cores were taken at 1.3 m from D (*n* = 5) and ND (*n* = 5) trees to measure wood anatomy. We measured two wood‐anatomical features (radial lumen diameter and double cell‐wall thickness) in the trees which showed the highest correlations of their individual ring‐width series with the mean site series. The 10‐mm cores were transversally cut using a sledge microtome (Gärtner et al. 2015) to obtain thin transversal sections (15–20 μm thick). Sections were mounted on glass slides, stained with safranin (0.5% in distilled water), Astra Blue (2%) and fixed with Eukitt. Images of sections were taken at 40–100x magnification with a digital camera mounted on a light microscope (Olympus BH2). They were stitched with the ICE software (Microsoft). Images were analysed for xylem measurement using the AutoCellRow software (Dyachuk et al. 2020). Lumen diameter and cell‐wall thickness were measured for each tracheid found along five radial rows of each annual ring. This was done for the period 2000−2020. We also calculated the thickness‐to‐span ratio as the square of the ratio of double wall thickness to lumen diameter (Hacke et al. 2001).

### 2.8 Pit Anatomy

We measured pit anatomy in stem and branch samples following Delzon et al. (2010) and Bouche et al. (2014). First, we took one 5‐mm core at 1.3 m from five D trees and five ND trees. Second, we analysed tangential earlywood cross‐sections of six 5‐year periods (1970−1974, 1980−1984, 1990−1994, 2000−2004, 2010−2014 and 2017−2021). Ten earlywood pits were measured in each period and tree. In addition, pit variables were measured in the earlywood of the 2001 ring in branches of ND and D trees (*n* = 50 pits per class type) used to obtain vulnerability curves. Pit anatomy was measured using images derived from scanning electron microscopy. The images were analysed using the image analysis freeware ImageJ ver. 1.54i (Schneider et al. 2012). Specifically, we measured the pit aperture diameter (Da), torus diameter (Dt) and pit membrane diameter (Dm). This allowed calculating the following variables (see Delzon et al. 2010):

(3) Torusoverlap=(Dt−Da)/Dt

(4) Margoflexibility=(Dm−Dt)/Dm

(5) Valveeffect=Torusoverlap×Margoflexibility

### 2.9 Concentrations of NSCs

To assess the availability of NSCs, we measured the concentrations of starch and soluble sugars (SS) in the stem sapwood. In early June 2020, sapwood cores were taken at 1.3 m using a Pressler increment borer from the tagged ND (*n* = 15) and D (*n* = 15) trees. The samples were placed in a cooler and taken immediately to the laboratory where they were freeze‐dried and milled to a fine powder in a Retsch M400 ball mill (Haan, Germany) before analysis. SS were extracted with 80% (v/v) ethanol in a water bath at 60°C, and their concentration was determined colourimetrically using the phenol–sulphuric method, as modified by Buysse and Merckx (1993). The starch and complex sugars that remained in the undissolved pellet after the ethanol extraction were enzymatically reduced to glucose using amyloglucosidase (0.5% amyloglucosidase 73.8 U/mg, Fluka 10115), and analysed following Palacio et al. (2007). NSCs measured after ethanol extraction are referred to as SSs, and those measured after enzymatic digestion are referred to as starch. The sum of SSs and starch is referred to as total NSC.

### 2.10 iWUE

We measured wood carbon isotopic composition (δ^13^C) to estimate iWUE. First, we selected the five D trees and the five ND trees whose ring‐width series showed the strongest correlations with the mean series of each vigour class. Then, we took 10‐mm cores and cut them transversally using a core microtome (Gärtner et al. 2015). We separated the annual rings from the cores using scalpels, focusing on the period from 2000 to 2020. Wood samples were then milled and homogenised using a ball mill (Retsch MM301, Haan, Germany), after which 0.8–1.2 mg aliquots were weighed into tin foil capsules using a microbalance (AX205 Mettler Toledo, OH, USA). Samples were combusted to CO_2_ using a Flash EA‐1112 elemental analyser interfaced with a Finnigan MAT Delta C isotope ratio mass spectrometer (Thermo Fisher Scientific Inc., MA, USA). Isotope analyses were conducted at the Stable Isotope Facility of the University of California at Davis (USA). Stable isotope ratios were expressed as per mil deviations using the δ notation relative to Vienna Pee Dee Belemnite. The standard deviation for repeated analyses was better than 0.1‰.

We calculated iWUE using the following framework. First, to account for changes in δ^13^C of atmospheric CO_2_ (*C*_*a*_), we calculated C isotope discrimination in wood (Δ^13^ *C*_*w*_) from δ^13^ *C*_*a*_ and wood δ^13^ *C*_*w*_ following Farquhar and Richards (1984):

(6) $$\Delta^{13}Cw=\left(\delta^{13}C_{a}-\delta^{13}C_{w}\right)/(1+\delta^{13}C_{w}/1000)$$

The *C*_*a*_ and δ^13^ *C*_*a*_ values were obtained from Belmecheri and Lavergne (2020). Second, we estimated iWUE (in μmol H_2_O mol^−1^ CO_2_) following Farquhar et al. (1982):

(7) $$\text{iWUE}\;=C_{a}\left[1–\left(C_{i}/C_{a}\right)\right]\;0.625$$

where *C*_*i*_ is the CO_2_ concentration in the sub‐stomatal cavity of leaves, and 0.625 is the relation among the conductance of water compared to the conductance of CO_2_. To determine *C*_*i*_ we used the equation proposed by Francey and Farquhar (1982):

(8) $$C_{i}=C_{a}(\delta^{13}C_{w}-\delta^{13}C_{a}+1)/(b-a)],$$

where *a* is the diffusion fractionation across the boundary layer and the stomata (+4.4‰) and *b* is the Rubisco fractionation (+27.0‰).

### 2.11 Statistical Analyses

Trends of seasonal climate variables were evaluated using Mann–Kendall tests. Then, the normality of the data was checked using the Shapiro–Wilk test. Variables that did not fulfil normality, such as BAI, were log (*x* + 1) transformed. To compare variables between the two tree classes and also within each sampling date, we used ANOVAs and post hoc Welch *t* tests. For pit anatomy data, we compared the values of ND and D trees for consecutive 5‐year periods. Finally, to compare the BAI series between ND and D trees, we used non‐parametric Wilcoxon rank–sum *W* tests.

## 3 Results

### 3.1 Tree Size, Defoliation and Mortality Data

All trees tagged as ND individuals remained alive from 2020 to 2024, except one (mean mortality rate was 1.33% year^−1^), whereas survival was lower in D trees, with 8 out of 15 trees dying during the same period (mean mortality rate was 10.7% year^−1^). The D trees showed smaller diameter (21.3 vs. 26.1 cm) and height (5.8 vs. 6.3 m) than ND trees (Table 1). Crown defoliation was consistently higher in D trees than in ND trees (84% vs. 21%, according to the 2024 data), but the soil depth was greater for ND trees (46 vs. 31 cm).

**Table 1:** Size (Dbh, diameter at breast height; height), age, soil depth and crown defoliation measured in declining (D) and non‐declining (ND) trees. Values are means ± SE. Different letters indicate significant differences between tree classes (*p* < 0.05).

Tree class	Dbh (cm)	Height (m)	Age at 1.3 m (years)	Soil depth (cm)	Crown defoliation (%)				
					2020	2021	2022	2023	2024
D	21.3 ± 0.7a	5.8 ± 0.2a	88 ± 2	31.1 ± 2.9a	58 ± 3b	75 ± 3b	79 ± 3b	80 ± 5b	84 ± 3b
ND	26.1 ± 0.9b	6.3 ± 0.1b	92 ± 2	45.8 ± 4.8b	11 ± 2a	18 ± 2a	19 ± 3a	22 ± 3a	21 ± 3a

### 3.2 Soil Isotopes

**Figure 1:**
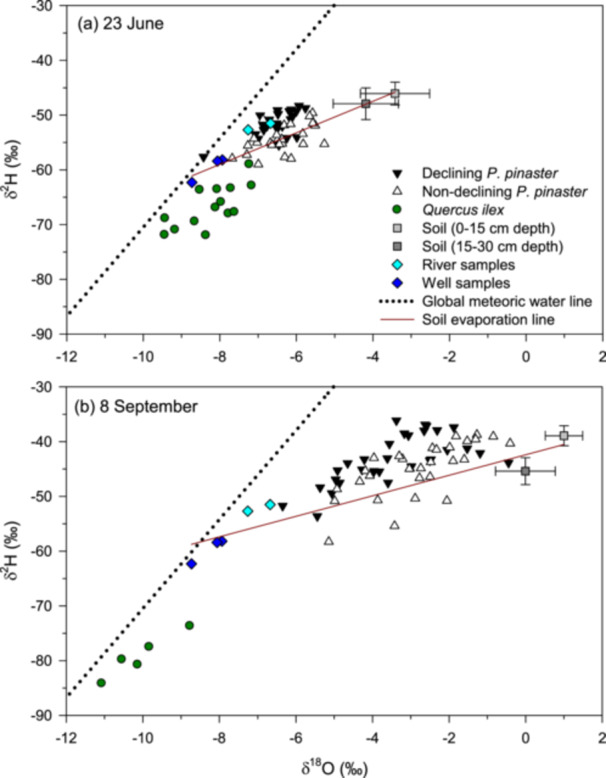
Isotope (δ^18^O, δ^2^H) measurements taken from water samples collected from xylem, soil, wells (dark blue diamonds) and a river (light blue diamonds) on two dates in 2020 (a, 23 June; b, 8 September). Xylem samples were obtained from *P. pinaster* trees in decline (black downward triangles) and non‐declining *P. pinaster* trees (upward white triangles) and *Q. ilex* trees (green circles). Soil samples were taken at 0–15 cm (light grey squares) and 15–30 cm (dark grey squares) depths. The dotted line shows the global meteoric water line, whereas the dark red line shows the soil evaporation line (based on river, well and soil samples).

According to soil and xylem water isotope (δ^18^O, δ^2^H) data, ND trees tended to extract water from deeper soil layers than D trees (i.e., more negative isotopic compositions in ND trees), whereas *Q. ilex* roots accessed the deepest soil water sources (Figures 1 and S5, Table S1). The difference in the depth of soil‐water sources between D and ND trees was greater in late summer than in spring. Furthermore, in both seasons, ND trees followed the soil evaporation line, which originates from the intersection of the isotopic compositions of water recharge sources (e.g., wells and river) and deep and shallow soil water, more closely than D trees. This suggests that ND trees used water from the same (i.e., current) meteoric origin, while D trees departed from the current soil evaporation line (Figure 1). In fact, in summer, D trees remained along the evaporation line of the soil of the previous (spring) sampling period.

### 3.3 Water Potential and Hydraulic Conductance

**Figure 2:**
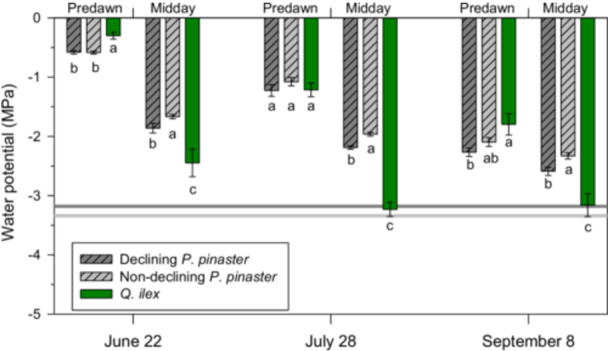
Water potential measurements carried out in declining (dark grey bars) and non‐declining (light grey bars) pine trees, and also on *Q. ilex* (green bars) from June to September 2020. Water potentials were measured at predawn and midday. The lower horizontal lines show the P_50_ values of declining trees (−3.18 MPa, dark grey horizontal line) and non‐declining trees (−3.34 MPa, light grey horizontal line). Values are means ± SE. Different letters indicate significant (*p* < 0.05) differences between tree classes within each sampling date. [Color figure can be viewed at wileyonlinelibrary.com]

**Figure 3:**
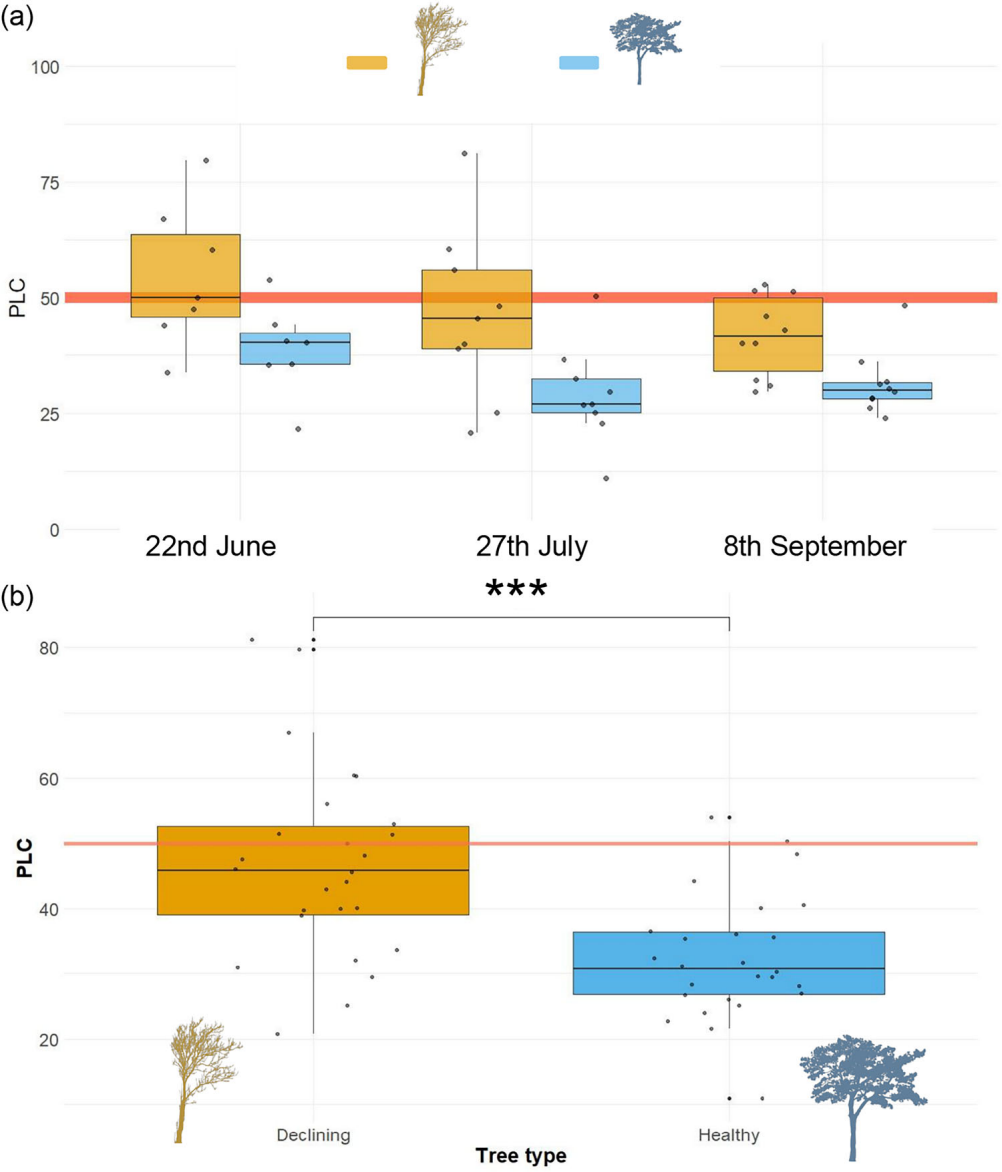
(a) Percent loss of conductance (PLC) values for the two tree classes and different sampling dates during 2020. (b) PLC values for declining and non‐declining trees showed significant differences between the two classes (***p < 0.001). Orange and blue box plots correspond to declining and non‐declining or healthy trees, respectively. The horizontal orange line shows the PLC threshold (50%) for mortality in conifers. [Color figure can be viewed at wileyonlinelibrary.com]

*Q. ilex* trees were able to reach lower water potentials (−3.20 MPa, midday, late July) than ND and D *P. pinaster* individuals (Figures 2, S6 and S7). The D trees reached significantly lower midday water potentials than the ND trees (−1.39 vs. −1.21 MPa; Table 2, Figure 2). The D trees showed significantly higher P_50_ values (−3.18 vs. −3.34 MPa) and *g*_*min*_ values than ND trees (0.84 vs. 0.64 mmol mol^−2^ s^−1^). Water potentials measured in the field in D and ND trees progressively declined during the summer, albeit did not reach the P_50_ thresholds (Figures 2, S6 and S7). Interestingly, we found significant negative correlations between late‐July midday water potential and crown defoliation or *gmin* (Figure S8). Lastly, the D trees showed higher PLC values than ND trees, with PLC values measured in late June and late July close to the threshold for mortality (P_50_) in conifers (Figure 3).

**Table 2:** Leaf variables measured in declining (D) and non‐declining (ND) trees. Data on water potential correspond to early June. Values are means ± SE. Different letters indicate significant differences between tree classes (*p* < 0.05).

Tree class	Predawn water potential (MPa)	Midday water potential (MPa)	Leaf area (cm^2^)	Leaf dry weight (g)	LMA (g m^−2^)	*g*_*min*_ (mmol m^−2^ s^−1^)
D	−0.52 ± 0.04	−1.39 ± 0.05a	91.20 ± 17.47	1.82 ± 0.33	211.14 ± 7.76	0.84 ± 0.08b
ND	−0.47 ± 0.02	−1.21 ± 0.06b	106.32 ± 10.32	2.28 ± 0.23	215.01 ± 6.58	0.64 ± 0.05a

### 3.4 Growth and Wood Anatomy

**Figure 4:**
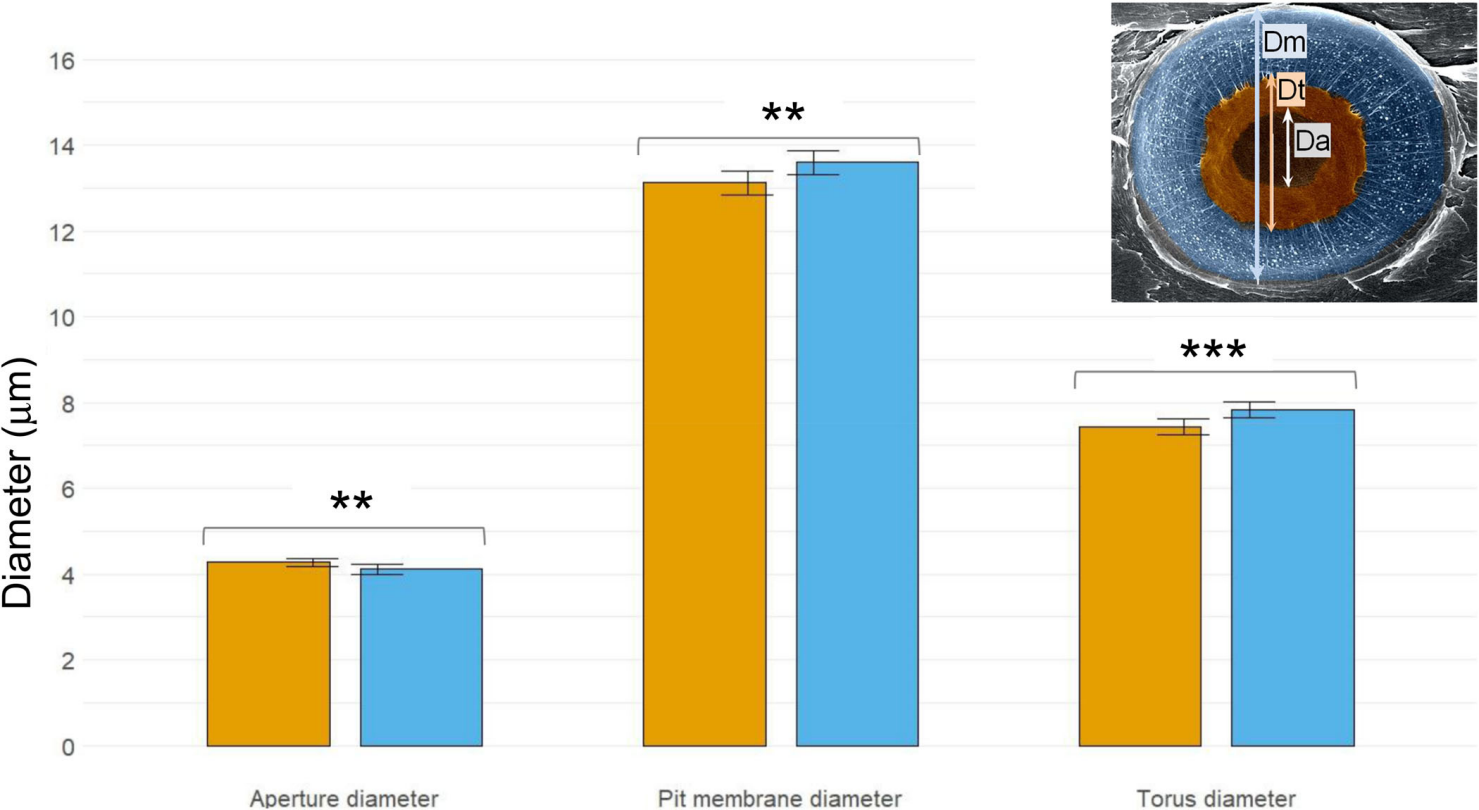
Pit anatomical measurements obtained for declining (orange bars, *n* = 10) and non‐declining (blue bars, *n* = 10) trees. The three variables are shown in the inset figure (Da, aperture diameter; Dt, torus diameter; Dm, pit membrane diameter; modified from Roskilly et al. 2019). Values are means ± SE. Asterisks indicate significant differences between tree classes (**p < 0.01; ***p < 0.01). [Color figure can be viewed at wileyonlinelibrary.com]

The D trees showed narrower rings and tracheids with smaller lumen diameter and thinner walls than ND trees (Table 3). In the branches, D trees formed pits with smaller pit membrane and torus diameters, but wider aperture diameter (Figure 4). D trees showed significantly lower values for torus overlap, margo flexibility and valve effect (Table 3).

**Table 3:** Growth and wood anatomy data were measured in the stems and branches of declining (D) and non‐declining (ND) trees. Pit anatomy data correspond to the earlywood formed in 2021. Values are means ± SE. Different letters indicate significant differences between tree classes (*p* < 0.05).

Tree class	Stem samples				Branch samples		
	Tree‐ring width (mm)	Lumen diameter (μm)	Cell‐wall thickness (μm^2^)	Thickness‐to‐span ratio (μm μm^−1^)	Torus overlap	Margo flexibility	Valve effect
D	0.37 ± 0.02a	21.5 ± 0.6a	9.34 ± 0.14a	0.20 ± 0.02	0.43 ± 0.01a	0.41 ± 0.01a	0.18 ± 0.01a
ND	0.45 ± 0.02b	24.3 ± 0.7b	9.78 ± 0.11b	0.17 ± 0.01	0.48 ± 0.01b	0.47 ± 0.02b	0.22 ± 0.01b

**Figure 5:**
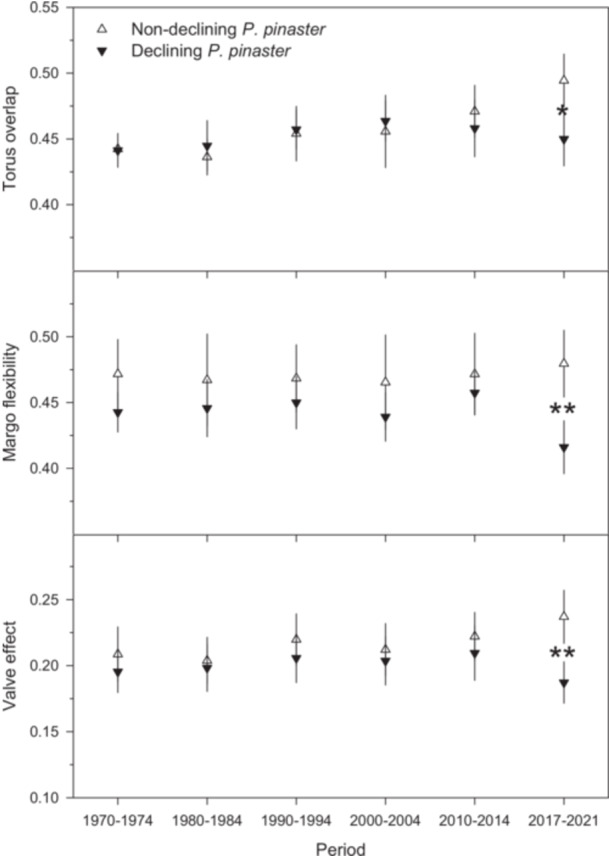
Pit anatomical measurements obtained for non‐declining (white symbols, *n* = 5) or declining (black symbols, *n* = 5) trees considering 5‐year periods. Significance levels: *p < 0.05; **p < 0.01. Values are means ± SE.

Differences in pit anatomy between D and ND trees varied over time in the analysed stem samples. In short, no significant differences were found between D and ND trees in terms of torus overlap, margo flexibility or valve effect between 1970 and 2014 (Figure 5). However, in the final study period (2017–2021), differences emerged, with D trees showing significantly lower values for all three variables: torus overlap (*t* = 2.60, *p* = 0.02), margo flexibility (*t* = 3.15, *p* = 0.007) and valve effect (*t* = 4.51, *p* = 0.007).

### 3.5 Stem Sapwood NSC Concentrations, Growth and iWUE

We found no significant differences in the sapwood concentrations of starch, SSs and NSCs between D and ND trees (Table S2).

**Figure 6:**
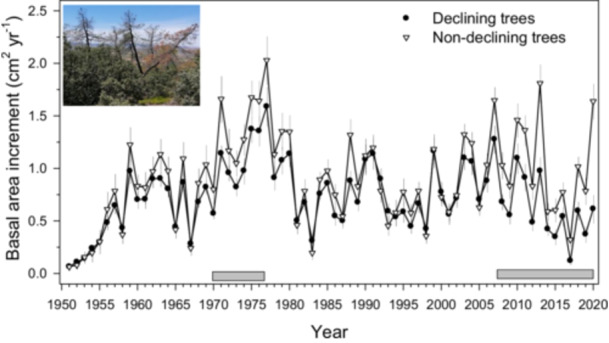
Series of basal area increment (BAI) calculated for declining (*n* = 30) and non‐declining (*n* = 30) trees. Grey boxes show periods when the BAI of declining trees was significantly (*p* < 0.05) higher than the BAI of ND trees according to Wilcoxon rank‐sum *W* tests. The inset shows recently dead pines. Values are means ± SE. [Color figure can be viewed at wileyonlinelibrary.com]

**Figure 7:**
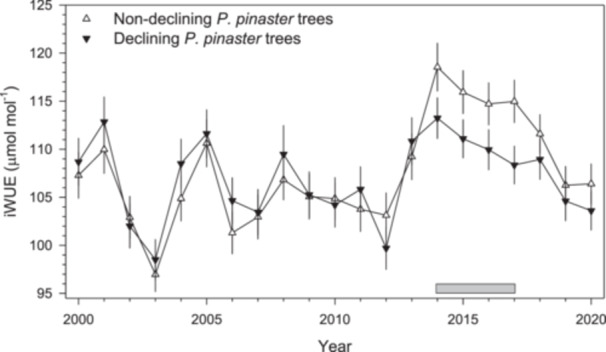
Values of intrinsic water‐use efficiency (iWUE) calculated for non‐declining (white symbols, *n* = 5) or declining (black symbols, *n* = 5) trees. The grey box indicates the period when iWUE values significantly (*p* < 0.05) differed between the two groups of trees. Values are means ± SE.

The D trees showed significantly lower BAI values than ND trees during the early 1970s, a wet‐cool period, and after 2007 when climate conditions turned warmer and more arid (Figure 6). The two vigour classes showed similar iWUE values, excepting from 2014 to 2017 when ND trees presented higher iWUE than D trees (Figure 7).

## 4 Discussion

Our results suggest that trees were predisposed to drought die‐off by: (i) accessing shallow soil water pools during the dry summer, (ii) forming tracheids with larger pit apertures, smaller torus overlap and valve effect, and (iii) presenting higher minimum leaf conductance (*g*_*min*_). Factor (i) explains a reduced capacity of vulnerable trees to access deep soil water and bedrock sources. Factor (ii) explains why declining trees showed higher levels of PLC and links hydraulic failure with defoliation, growth decline and tree death. Finally, factor (iii) confirms that vulnerable trees are prone to higher residual water losses, that is, after stomatal closure, through leaves.

Some of the proposed hypotheses and expectations were supported by our findings, whereas others were not (Table 4). First, the impact of drought on tree vigour, as assessed by crown defoliation, was predisposed by a lower capacity to access deep soil water sources during the dry late summer period. This is consistent with other studies in Mediterranean forests (Colangelo et al. 2017; Ripullone et al. 2020). It suggests that trees that did not decline formed a deeper and more developed or efficient root system. This could be linked to their greater height and diameter. Furthermore, the declining trees decoupled from soil values in summer and followed an evaporation line similar to that of the soil in spring. This indicates an uncoupling between soil and xylem water in declining pines due to limited transpiration (Del Castillo et al. 2016). The isotopic enrichment of xylem water in declining pines from spring to late summer could be a consequence of evaporative enrichment in the stem due to water stagnation during periods of restricted sap flow (Dawson and Ehleringer 1993; Körner 2019). In contrast, oaks, which showed minimal defoliation, were able to uptake deeper soil water than pines, as observed in other Mediterranean pine‐oak forests (Martín‐Gómez, Aguilera, et al. 2017). Although *Q. ilex* may show much lower P_50_ values (up to −7.13 MPa), and thus be more resistant to xylem embolism, than coexisting pine species (Peguero‐Pina et al. 2014), this oak species can also suffer dieback in sites with shallow and rocky soils (Gea‐Izquierdo et al. 2025). Therefore, studies on root functioning, including conductance measurements and soil‐root interactions, are needed to improve our understanding of the mechanisms of forest die‐off. Second, declining trees showed lower radial growth rates, formed tracheids with smaller lumens and thinner walls. Severe droughts also caused high mortality of resin‐tapped *P. pinaster* forests in central Spain (Calama et al. 2024). However, our stand was not formerly tapped, and the tree‐ring series did not show releases, suggesting that thinning did not affect the study stand at least since the 1950s. The observed xylem anatomical features lead to a lower theoretical hydraulic conductance. Importantly, pit anatomy data confirm hydraulic dysfunction because declining trees have smaller torus overlap and valve effect due to larger pit apertures, which should lead to a lower efficiency in avoiding the air passing between tracheids and, therefore, to a higher vulnerability to drought‐induced xylem cavitation (Delzon et al. 2010; Bouche et al. 2014). This would explain the higher levels of embolism formation observed in declining trees under less negative water potentials than for the non‐declining trees. Note also that our water potentials only corresponded to 3 days in summer, and lower values could have been reached on other dates. Interestingly, the differences in growth and pit anatomy emerged in the most recent period, indicating that the severe 2010s droughts (e.g., 2017) led to the crossing of physiological ‘no‐return’ thresholds (Hammond et al. 2019). Third, declining trees showed a higher minimum leaf conductance, indicating a higher canopy water loss and more rapid dehydration. The recent droughts were characterised by elevated maximum summer temperatures during several years, which probably caused exponential rises of VPD, and triggered hydraulic failure, causing tree death (Cochard 2021; Petek‐Petrik et al. 2023). In woody plants, a timely stomatal closure and late xylem embolism onset are frequently observed in xeric sites (Jin et al. 2023). However, hot droughts can challenge these adaptations, as elevated temperatures increase *g*_*min*_, thereby exacerbating the negative effects of water shortage, even if dying or surviving trees form leaves of similar size.

**Table 4:** Summary of the expectations and results found in the comparisons between declining (D) and non‐declining (ND) trees.

Process, variable or organ	Variable or trait	Expectation	Finding
Depth of soil water uptake	Water H and O isotopes	D < ND	✓
Soil depth	Direct measurement	D < ND	✓
Tree size	Diameter (dbh)	D < ND	✓
	Height	D < ND	✓
Radial growth	Basal area increment	D < ND	✓
Wood anatomy	Lumen area	D < ND	✓
	Cell‐wall thickness	D < ND	✓
	Thickness‐to‐span ratio	D < ND	✗
	Pit aperture diameter	D > ND	✓
	Valve effect	D < ND	✓
	Torus overlap	D < ND	✓
Hydraulics	PLC	D > ND	✓
	P_50_	D > ND	✓
Water‐carbon balance	iWUE	D < ND	✗
Carbon reserves	NSC concentrations	D < ND	✗
Leaf and shoot	Leaf area	D < ND	✗
	Leaf dry weight	D < ND	✗
	Midday water potential	D < ND	✓
	*g*_*min*_	D > ND	✓

We found anatomical and functional variables differentiating the two vigour classes and supporting the major roles played by pit aperture and *g*_*min*_ as drivers of tree death. However, our findings did not support expectations based on the current paradigms driving tree drought mortality and related to carbon starvation (Table 4). Water shortage limits growth before a reduction in photosynthesis occurs (Körner 2015). Thus, drought increases water‐use efficiency and leads to an accumulation of NSCs through a decrease in growth rates (Oberhuber et al. 2011; Dietze et al. 2014; Martínez‐Sancho et al. 2022). However, we did not find different sapwood NSC concentrations between the two vigour classes. This could be explained by the fact that differences in NSCs would have been found in other organs, such as roots, which depend on the transport of mobile sugars through the phloem (Hartmann et al. 2013; Li et al. 2018). Lastly, declining trees did not show higher iWUE during the most recent dry period, which suggests a poor ability to control water loss (higher stomatal conductance rate). Such a water‐spender strategy has been observed in other studies of declining Scots pine (*Pinus sylvestris*) trees, which also presented high growth rates and formed wider tracheids (Voltas et al. 2013), but this was not the case, suggesting a chronic stressful situation in the study stand. A dual‐isotope approach (δ^13^C, δ^18^O) could help to disentangle the relative roles played by photosynthesis and stomatal conductance rates on this relative loss of iWUE. As has been observed in silver fir (*Abies alba*), an improved iWUE does not avoid the hydraulic deterioration before tree death (Linares and Camarero 2012; Pellizzari et al. 2016).

Fine‐scale environmental differences between coexisting trees, such as different soil depths or rock cover, could explain why declining trees were more negatively impacted by drought stress. For instance, in a drought‐affected Aleppo pine (*Pinus halepensis*) forest, a higher surface rock cover and stoniness across the soil profile result in higher soil water availability and buffer long dry seasons (Preisler et al. 2019). We cannot rule out that declining trees were found in poorer microsites, albeit no differences in soil (nutrients, microbiome composition and biomass) were found between the two vigour classes (Gazol et al. 2024), and soil water uptake depth may also influence the nutritional status of trees (González de Andrés et al. 2022). Studies on tree‐microsite linkages could be complemented with landscape analyses based on drone images (e.g., Flynn et al. 2024) to assess whether declining trees are more abundant on rocky sites in drier locations (e.g., S‐SE exposure, higher slope). Genetic differences between conspecific individuals could also explain their different responses to drought stress. In *P. pinaster*, there is a high inter‐ and intra‐population genetic variation for some traits providing drought tolerance (Corcuera et al. 2012; Velasco‐Conde et al. 2012). However, different provenances of *P. pinaster* did not vary in cavitation resistance, suggesting low genetic variation or uniform selection (Lamy et al. 2011). Therefore, genetic differences within the study population do not seem to be a plausible explanation for drought‐triggered vigour differences between neighbouring trees.

Finally, our results have clear implications for forecasting the vulnerability of conifer forests to hotter droughts. Trees uptaking shallow soil water sources, forming tracheids with large pit apertures, and showing high *g*_*min*_ will be the most vulnerable to soil water shortage (reduced water uptake, higher risk of xylem cavitation) and rising atmospheric water demand (excessive water loss). Some tree species, such as *P. pinaster,* seem to be particularly vulnerable to such hot‐drought stress and can be considered as a ‘lookout’ or early‐warning species.

## 5 Conclusions

Our findings on *P. pinaster* provide support for hydraulic failure as a major cause of tree drought mortality because declining trees were more vulnerable to xylem embolism due to wood anatomical (large pit aperture, small torus overlap and valve effect) and leaf functional traits (higher *g*_*min*_). However, we also found that declining trees were less able to access deep soil water sources during the dry summer than non‐declining trees. The combination of these factors disrupts the soil‐plant‐atmosphere continuum by interrupting water transport through the xylem, enhancing leaf water loss and constraining soil water uptake.

## Supporting information


**Figure S1:** (a) Climate diagram and (b) monthly water budgets based on monthly climate data from the Daroca station (period 1920–2018).
**Figure S2:** Seasonal climate data measured in the Daroca station. Lines indicate linear regression for variables showing significant (*p* < 0.05) trends according to Mann‐Kendall tests.
**Figure S3:** Histogram showing historical data of annual climatic water balances (Daroca station, period 1920–2018) and the balance recorded in the severe 2017 drought (dotted vertical line).
**Figure S4:** Daily climate data recorded during the monitoring period (2016–2020) when most measurements of wood anatomy, leaf variables, water potential and hydraulic conductivity were done. Plots show (a) mean maximum temperatures, (b) mean minimum temperatures, and (c) total precipitation. Data are from Daroca meteorological station and lines show mean values for annual cycles (period 1920–2020).
**Figure S5:** Differences in the isotope (δ^18^O, δ^2^H) measurements made in water samples taken from xylem and soils during spring (23^rd^ June) and late summer (8^th^ September) in 2020. Xylem samples were obtained from declining (downward black triangles) and non‐declining *P. pinaster* (upward white triangles) and from *Q. ilex* trees (green circles). Soil samples were taken at 0–15 cm (light grey squares) and 15–30 cm (dark grey squares) depths.
**Figure S6:** Relationships between water potential and specific hydraulic conductivity (K_s_) in declining (orange symbols and lines) and non‐declining (blue symbols and lines) trees. Sampling date: June 2020.
**Figure S7:** Relationships between water potential and percent loss of conductivity (PLC) in declining (orange symbols and lines) and non‐declining (blue symbols and lines) trees. The horizontal dashed line shows the water potentials at which 50 % of hydraulic conductivity is lost (P50). Sampling date: June 2020. The P50 value of declining trees (3.18 MPa) was significantly (*p* < 0.05) lower than that measured in non‐declining trees (3.34 MPa).
**Figure S8:** Significant negative correlations (Pearson coefficients) found between mean (a) crown defoliation or (b) *g_min_* and late‐July midday water potential. Variables were measured in 2020.
**Table S1:** Data on xylem and soil water isotopes measured in late June and early September 2020. Values are means ± SE. The last lines show *F* statistic corresponding to one‐way ANOVAs between the three groups of trees, whereas different letters indicate significant (*p* < 0.05) differences between groups (*t* tests).
**Table S2:** Concentrations of NSCs measured in sapwood samples. Values are means ± SE. Different letters indicate significant (*p* < 0.05) differences between groups of trees (*t* tests).

## Data Availability

The data that support the findings of this study are available on request from the corresponding author. The data are not publicly available due to privacy or ethical restrictions.

## References

[pce70211-bib-0001] Adams, H. D. , M. J. B. Zeppel , W. R. L. Anderegg , et al. 2017. “A Multi‐Species Synthesis of Physiological Mechanisms in Drought‐Induced Tree Mortality.” Nature Ecology & Evolution 1: 1285–1291. 10.1038/s41559-017-0248-x. 29046541

[pce70211-bib-0003] Allen, C. D. , A. K. Macalady , H. Chenchouni , et al. 2010. “A Global Overview of Drought and Heat‐Induced Tree Mortality Reveals Emerging Climate Change Risks for Forests.” Forest Ecology and Management 259: 660–684.

[pce70211-bib-0002] Allen, C. D. , D. D. Breshears , and N. G. McDowell . 2015. “On Underestimation of Global Vulnerability to Tree Mortality and Forest Die‐Off From Hotter Drought in the Anthropocene.” Ecosphere 6: 1–55.

[pce70211-bib-0004] Anderegg, W. R. L. , T. Klein , M. Bartlett , et al. 2016. “Meta‐Analysis Reveals That Hydraulic Traits Explain Cross‐Species Patterns of Drought‐Induced Tree Mortality Across the Globe.” Proceedings of the National Academy of Sciences 113: 5024–5029. 10.1073/pnas.1525678113. PMC4983847 27091965

[pce70211-bib-0005] Arend, M. , R. M. Link , R. Patthey , G. Hoch , B. Schuldt , and A. Kahmen . 2021. “Rapid Hydraulic Collapse as Cause of Drought‐Induced Mortality in Conifers.” Proceedings of the National Academy of Sciences 118: e2025251118. 10.1073/pnas.2025251118. PMC8072240 33846261

[pce70211-bib-0008] Belmecheri, S. , and A. Lavergne . 2020. “Compiled Records of Atmospheric CO_2_ Concentrations and Stable Carbon Isotopes to Reconstruct Climate and Derive Plant Ecophysiological Indices From Tree Rings.” Dendrochronologia 63: 125748. 10.1016/j.dendro.2020.125748.

[pce70211-bib-0009] Blackman, C. J. , L.‐M. Billon , J. Cartailler , J. M. Torres‐Ruiz , and H. Cochard . 2023. “Key Hydraulic Traits Control the Dynamics of Plant Dehydration in Four Contrasting Tree Species During Drought.” Tree Physiology 43: 1772–1783. 10.1093/treephys/tpad075. 37318310 PMC10652334

[pce70211-bib-0011] Bouche, P. S. , M. Larter , J.‐C. Domec , et al. 2014. “A Broad Survey of Hydraulic and Mechanical Safety in the Xylem of Conifers.” Journal of Experimental Botany 65: 4419–4431. 10.1093/jxb/eru218. 24916072 PMC4112641

[pce70211-bib-0010] Bouche, P. S. , S. Delzon , B. Choat , et al. 2016. “Are Needles of *Pinus pinaster* More Vulnerable to Xylem Embolism Than Branches? New Insights From X‐Ray Computed Tomography.” Plant, Cell & Environment 39: 860–870. 10.1111/pce.12680. 26574193

[pce70211-bib-0012] Brodribb, T. J. , and H. Cochard . 2009. “Hydraulic Failure Defines the Recovery and Point of Death in Water‐Stressed Conifers.” Plant Physiology 149: 575–584. 19011001 10.1104/pp.108.129783 PMC2613726

[pce70211-bib-0013] Brodribb, T. J. , J. Powers , H. Cochard , and B. Choat . 2020. “Hanging by a Thread? Forests and Drought.” Science 368: 261–266. 32299945 10.1126/science.aat7631

[pce70211-bib-0014] Bueno, A. , D. Alonso‐Forn , J. J. Peguero‐Pina , et al. 2022. “Minimum Leaf Conductance (*g* _ *min* _) Is Higher in the Treeline of *Pinus uncinata* Ram. in the Pyrenees: Michaelis’ Hypothesis Revisited.” Frontiers in Plant Science 12: 786933. 10.3389/fpls.2021.786933. 35140730 PMC8818696

[pce70211-bib-0015] Bueno, A. , D. Sancho‐Knapik , E. Gil‐Pelegrín , et al. 2020. “Cuticular Wax Coverage and Its Transpiration Barrier Properties in *Quercus coccifera* L. Leaves: Does the Environment Matter?” Tree Physiology 40: 827–840. 10.1093/treephys/tpz110. 31728539

[pce70211-bib-0016] Bunn, A. G. 2008. “A Dendrochronology Program Library in R (dplR).” Dendrochronologia 26: 115–124. 10.1016/j.dendro.2008.01.002.

[pce70211-bib-0017] Bunn, A. G. 2010. “Statistical and Visual Crossdating in R Using the dplR Library.” Dendrochronologia 28: 251–258. 10.1016/j.dendro.2009.12.001.

[pce70211-bib-0018] Bunn, A. G. , M. Korpela , F. Biondi , et al. 2024. dplR: Dendrochronology Program Library in R. R Package Version 1.7.7. https://CRAN.R-project.org/package=dplR.

[pce70211-bib-0019] Burghardt, M. 2003. “Ecophysiological Relevance of Cuticular Transpiration of Deciduous and Evergreen Plants in Relation to Stomatal Closure and Leaf Water Potential.” Journal of Experimental Botany 54: 1941–1949. 12815029 10.1093/jxb/erg195

[pce70211-bib-0020] Buysse, J. , and R. Merckx . 1993. “An Improved Colorimetric Method to Quantify Sugar Content of Plant Tissue.” Journal of Experimental Botany 44: 1627–1629.

[pce70211-bib-0021] Calama, R. , C. Martínez , J. Gordo , M. Del Río , M. Menéndez‐Miguélez , and M. Pardos . 2024. “The Impact of Climate and Management on Recent Mortality in *Pinus pinaster* Resin‐Tapped Forests of Inland Spain.” Forestry 97: 120–132. 10.1093/forestry/cpad023.

[pce70211-bib-0023] Camarero, J. J. , A. Gazol , G. Sangüesa‐Barreda , J. Oliva , and S. M. Vicente‐Serrano . 2015. “To Die or Not to Die: Early Warnings of Tree Dieback in Response to a Severe Drought.” Journal of Ecology 103: 44–57.

[pce70211-bib-0022] Camarero, J. J. , M. Colangelo , A. Gazol , E. González de Andrés , and C. Valeriano . 2025. “Lower Growth and Production of Latewood Intra‐Annual Density Fluctuations Due to Drought‐Triggered Forest Die‐Off.” Trees, Forests and People 20: 100843.

[pce70211-bib-0025] Cochard, H. 2021. “A New Mechanism for Tree Mortality Due to Drought and Heatwaves.” Peer Community Journal 1: e36. 10.24072/pcjournal.45.

[pce70211-bib-0026] Cochard, H. , G. Damour , C. Bodet , I. Tharwat , M. Poirier , and T. Améglio . 2005. “Evaluation of a New Centrifuge Technique for Rapid Generation of Xylem Vulnerability Curves.” Physiologia Plantarum 124: 410–418.

[pce70211-bib-0027] Colangelo, M. , J. J. Camarero , M. Borghetti , A. Gazol , T. Gentilesca , and F. Ripullone . 2017. “Size Matters a Lot: Drought‐Affected Italian Oaks Are Smaller and Show Lower Growth Prior to Tree Death.” Frontiers in Plant Science 8: 135. 10.3389/fpls.2017.00135. 28270816 PMC5318376

[pce70211-bib-0028] Corcuera, L. , E. Gil‐Pelegrin , and E. Notivol . 2012. “Differences in Hydraulic Architecture Between Mesic and Xeric *Pinus pinaster* Populations at the Seedling Stage.” Tree Physiology 32: 1442–1457. 10.1093/treephys/tps103. 23148035

[pce70211-bib-0029] Craig, H. 1961. “Isotopic Variations in Meteoric Waters.” Science 133: 1702–1703. 17814749 10.1126/science.133.3465.1702

[pce70211-bib-0030] Dawson, T. E. , and J. R. Ehleringer . 1993. “Isotopic Enrichment of Water in the Woody Tissues of Plants—Implications for Plant Water Source, Water‐Uptake, and Other Studies Which Use the Stable Isotopic Composition of Cellulose.” Geochimica et Cosmochimica Acta 57: 3487–3492.

[pce70211-bib-0024] Del Castillo, J. , C. Comas , J. Voltas , and J. P. Ferrio . 2016. “Dynamics of Competition Over Water in a Mixed Oak‐Pine Mediterranean Forest: Spatio‐Temporal and Physiological Components.” Forest Ecology and Management 382: 214–224. 10.1016/j.foreco.2016.10.025.

[pce70211-bib-0031] Delzon, S. , C. Douthe , A. Sala , and H. Cochard . 2010. “Mechanism of Water‐Stress Induced Cavitation in Conifers: Bordered Pit Structure and Function Support the Hypothesis of Seal Capillary‐Seeding.” Plant, Cell & Environment 33: 2101–2111. 10.1111/j.1365-3040.2010.02208.x PMC3003904 20636490

[pce70211-bib-0032] Dietze, M. C. , A. Sala , M. S. Carbone , et al. 2014. “Nonstructural Carbon in Woody Plants.” Annual Review of Plant Biology 65: 667–687. 10.1146/annurev-arplant-050213-040054 24274032

[pce70211-bib-0033] Duursma, R. A. , C. J. Blackman , R. Lopéz , N. K. Martin‐StPaul , H. Cochard , and B. E. Medlyn . 2019. “On the Minimum Leaf Conductance: Its Role in Models of Plant Water Use, and Ecological and Environmental Controls.” New Phytologist 221: 693–705. 10.1111/nph.15395. 30144393

[pce70211-bib-0101] Dyachuk, P. , A. Arzac , P. Peresunko , et al. 2020. “AutoCellRow (ACR) – A New Tool for the Automatic Quantification of Cell Radial Files in Conifer Images.” Dendrochronologia 60: 125687. 10.1016/j.dendro.2020.125687.

[pce70211-bib-0034] Eichhorn, J. , P. Roskams , N. Potočić , V. Timmermann , and M. Ferretti . 2016. “Part IV: Visual Assessment of Crown Condition and Damaging Agents.” In Manual on Methods and Criteria for Harmonized Sampling, Assessment, Monitoring and Analysis of the Effects of Air Pollution on Forests., edited by UNECE ICP Forests Programme Co‐Ordinating Centre. Thünen Institute of Forest Ecosystems.

[pce70211-bib-0036] Farquhar, G. , and R. Richards . 1984. “Isotopic Composition of Plant Carbon Correlates With Water‐Use Efficiency of Wheat Genotypes.” Functional Plant Biology 11: 539–552.

[pce70211-bib-0035] Farquhar, G. , M. O'Leary , and J. Berry . 1982. “On the Relationship Between Carbon Isotope Discrimination and the Intercellular Carbon Dioxide Concentration in Leaves.” Functional Plant Biology 9: 121–127.

[pce70211-bib-0037] Flynn, W. R. M. , S. W. D. Grieve , A. J. Henshaw , et al. 2024. “UAV‐Derived Greenness and Within‐Crown Spatial Patterning Can Detect Ash Dieback in Individual Trees.” Ecological Solutions and Evidence 5: e12343. 10.1002/2688-8319.12343.

[pce70211-bib-0038] Francey, R. J. , and G. D. Farquhar . 1982. “An Explanation of ^13^C/^12^C Variations in Tree Rings.” Nature 297: 28–31.

[pce70211-bib-0039] Fritts, H. C. 2012. Tree Rings and Climate. Academic Press.

[pce70211-bib-0040] Gärtner, H. , S. Lucchinetti , and F. H. Schweingruber . 2015. “A New Sledge Microtome to Combine Wood Anatomy and Tree‐Ring Ecology.” IAWA Journal 36: 452–459. 10.1163/22941932-20150114.

[pce70211-bib-0041] Gazol, A. , and J. J. Camarero . 2022. “Compound Climate Events Increase Tree Drought Mortality Across European Forests.” Science of the Total Environment 816: 151604. 34780817 10.1016/j.scitotenv.2021.151604

[pce70211-bib-0042] Gazol, A. , E. González de Andrés , Á. Valverde , J. M. Igual , A. Serrano , and J. J. Camarero . 2024. “The Strong Seasonality of Soil Microbial Community Structure in Declining Mediterranean Pine Forests Depends More on Soil Conditions Than on Tree Vitality.” Science of the Total Environment 957: 177560. 39557170 10.1016/j.scitotenv.2024.177560

[pce70211-bib-0043] Gea‐Izquierdo, G. , M. Férriz , M. Conde , M. N. Evans , J. I. Querejeta , and D. Martin‐Benito . 2025. “Die‐Off After an Extreme Hot Drought Affects Trees With Physiological Performance Constrained by a More Stressful Abiotic Niche.” Agricultural and Forest Meteorology 363: 110430. 10.1016/j.agrformet.2025.110430.

[pce70211-bib-0044] González de Andrés, E. , A. Gazol , J. I. Querejeta , et al. 2022. “The Role of Nutritional Impairment in Carbon‐Water Balance of Silver Fir Drought‐Induced Dieback.” Global Change Biology 28: 4439–4458. 10.1111/gcb.16170. 35320604 PMC9540818

[pce70211-bib-0045] Grossiord, C. , S. Sevanto , T. E. Dawson , et al. 2017. “Warming Combined With More Extreme Precipitation Regimes Modifies the Water Sources Used by Trees.” New Phytologist 213: 584–596. 27612306 10.1111/nph.14192

[pce70211-bib-0046] Hacke, U. G. , J. S. Sperry , W. T. Pockman , S. D. Davis , and K. A. McCulloh . 2001. “Trends in Wood Density and Structure Are Linked to Prevention of Xylem Implosion by Negative Pressure.” Oecologia 126: 457–461. 28547229 10.1007/s004420100628

[pce70211-bib-0047] Hammond, W. M. , A. P. Williams , J. T. Abatzoglou , et al. 2022. “Global Field Observations of Tree Die‐Off Reveal Hotter‐Drought Fingerprint for Earth's Forests.” Nature Communications 13: 1761. 10.1038/s41467-022-29289-2 PMC8983702 35383157

[pce70211-bib-0048] Hammond, W. M. , K. Yu , L. A. Wilson , R. E. Will , W. R. L. Anderegg , and H. D. Adams . 2019. “Dead or Dying? Quantifying the Point of No Return From Hydraulic Failure in Drought‐Induced Tree Mortality.” New Phytologist 223: 1834–1843. 31087656 10.1111/nph.15922 PMC6771894

[pce70211-bib-0049] Hartmann, H. , W. Ziegler , and S. Trumbore . 2013. “Lethal Drought Leads to Reduction in Nonstructural Carbohydrates in Norway Spruce Tree Roots but Not in the Canopy.” Functional Ecology 27: 413–427. 10.1111/1365-2435.12046.

[pce70211-bib-0050] Holmes, R. L. 1983. “Computer‐Assisted Quality Control in Tree‐Ring Dating and Measurement.” Tree‐Ring Bull 43: 68–78.

[pce70211-bib-0051] Jin, Y. , G. Hao , W. M. Hammond , et al. 2023. “Aridity‐Dependent Sequence of Water Potentials for Stomatal Closure and Hydraulic Dysfunctions in Woody Plants.” Global Change Biology 29: 2030–2040. 10.1111/gcb.16605. 36655297

[pce70211-bib-0052] Körner, C. 1995. “Leaf Diffusive Conductances in the Major Vegetation Types of the Globe.” In Ecophysiology of Photosynthesis, edited by E. D. Schulze and M. M. Caldwell , 463–490. Springer.

[pce70211-bib-0053] Körner, C. 2015. “Paradigm Shift in Plant Growth Control.” Current Opinion in Plant Biology 25: 107–114. 26037389 10.1016/j.pbi.2015.05.003

[pce70211-bib-0054] Körner, C. 2019. “No Need for Pipes When the Well Is Dry—A Comment on Hydraulic Failure in Trees.” Tree Physiology 39: 695–700. 10.1093/treephys/tpz030. 30938423

[pce70211-bib-0055] Lamy, J.‐B. , L. Bouffier , R. Burlett , C. Plomion , H. Cochard , and S. Delzon . 2011. “Uniform Selection as a Primary Force Reducing Population Genetic Differentiation of Cavitation Resistance Across a Species Range.” PLoS One 6: e23476. 21858137 10.1371/journal.pone.0023476 PMC3155568

[pce70211-bib-0058] Li, W. , H. Hartmann , H. D. Adams , et al. 2018 November 1. “The Sweet Side of Global Change‐Dynamic Responses of Non‐Structural Carbohydrates to Drought, Elevated CO_2_ and Nitrogen Fertilization in Tree Species.” Tree Physiology 38: 1706–1723. 10.1093/treephys/tpy059. 29897549

[pce70211-bib-0059] Linares, J. C. , and J. J. Camarero . 2012. “From Pattern to Process: Linking Intrinsic Water‐Use Efficiency to Drought‐Induced Forest Decline.” Global Change Biology 18: 1000–1015.

[pce70211-bib-0061] Mantova, M. , P. E. Menezes‐Silva , E. Badel , H. Cochard , and J. M. Torres‐Ruiz . 2021. “The Interplay of Hydraulic Failure and Cell Vitality Explains Tree Capacity to Recover From Drought.” Physiologia Plantarum 172: 247–257. 33432594 10.1111/ppl.13331

[pce70211-bib-0060] Mantova, M. , S. Herbette , H. Cochard , and J. M. Torres‐Ruiz . 2022. “Hydraulic Failure and Tree Mortality: From Correlation to Causation.” Trends in Plant Science 27: 335–345. 34772610 10.1016/j.tplants.2021.10.003

[pce70211-bib-0063] Martín‐Gómez, P. , A. Barbeta , J. Voltas , et al. 2015. “Isotope Ratio Infrared Spectroscopy: A Reliable Tool for the Investigation of Plant‐Water Sources?” New Phytologist 207: 914–927. 25790288 10.1111/nph.13376

[pce70211-bib-0064] Martín‐Gómez, P. , L. Serrano , and J. P. Ferrio . 2017. “Short‐Term Dynamics of Evaporative Enrichment of Xylem Water in Woody Stems: Implications for Ecohydrology.” Tree Physiology 37: 511–522. 27974650 10.1093/treephys/tpw115

[pce70211-bib-0062] Martín‐Gómez, P. , M. Aguilera , J. Pemán , E. Gil‐Pelegrín , and J. P. Ferrio . 2017. “Contrasting Ecophysiological Strategies Related to Drought: The Case of a Mixed Stand of Scots Pine (*Pinus sylvestris*) and a Submediterranean Oak (*Quercus subpyrenaica*).” Tree Physiology 37: 1478–1492. 29040771 10.1093/treephys/tpx101

[pce70211-bib-0065] Martin‐StPaul, N. , S. Delzon , and H. Cochard . 2017. “Plant Resistance to Drought Depends on Timely Stomatal Closure.” Ecology Letters 20: 1437–1447. 28922708 10.1111/ele.12851

[pce70211-bib-0066] Martínez‐Sancho, E. , K. Treydte , M. M. Lehmann , A. Rigling , and P. Fonti . 2022. “Drought Impacts on Tree Carbon Sequestration and Water Use‐Evidence From Intra‐Annual Tree‐Ring Characteristics.” New Phytologist 236: 58–70. 35576102 10.1111/nph.18224 PMC9542003

[pce70211-bib-0067] Maxwell, R. S. , and L. A. Larsson . 2021. “Measuring Tree‐Ring Widths Using the Coorecorder Software Application.” Dendrochronologia 67: 125841.

[pce70211-bib-0069] McDowell, N. G. , G. Sapes , A. Pivovaroff , et al. 2022. “Mechanisms of Woody‐Plant Mortality Under Rising Drought, Co_2_ and Vapour Pressure Deficit.” Nature Reviews Earth & Environment 3: 294–308. 10.1038/s43017-022-00272-1.

[pce70211-bib-0068] McDowell, N. , W. T. Pockman , C. D. Allen , et al. 2008. “Mechanisms of Plant Survival and Mortality During Drought: Why Do Some Plants Survive While Others Succumb to Drought?” New Phytologist 178: 719–739. 18422905 10.1111/j.1469-8137.2008.02436.x

[pce70211-bib-0071] Moreno‐Fernández, D. , A. Viana‐Soto , J. J. Camarero , M. A. Zavala , J. Tijerín , and M. García . 2021. “Using Spectral Indices as Early Warning Signals of Forest Dieback: The Case of Drought‐Prone *Pinus pinaster* Forests.” Science of the Total Environment 793: 148578. 34174606 10.1016/j.scitotenv.2021.148578

[pce70211-bib-0070] Moreno‐Fernández, D. , J. J. Camarero , M. García , et al. 2022. “The Interplay of the Tree and Stand‐Level Processes Mediate Drought‐Induced Forest Dieback: Evidence From Complementary Remote Sensing and Tree‐Ring Approaches.” Ecosystems 25: 1738–1753. 10.1007/s10021-022-00793-2.

[pce70211-bib-0072] Oberhuber, W. , I. Swidrak , D. Pirkebner , and A. Gruber . 2011. “Temporal Dynamics of Nonstructural Carbohydrates and Xylem Growth in *Pinus sylvestris* Exposed to Drought.” Canadian Journal of Forest Research 41: 1590–1597. 10.1139/x11-085 PMC3191854 22003262

[pce70211-bib-0073] Palacio, S. , M. Maestro , and G. Montserratmarti . 2007. “Seasonal Dynamics of Non‐Structural Carbohydrates in Two Species of Mediterranean Sub‐Shrubs With Different Leaf Phenology.” Environmental and Experimental Botany 59: 34–42.

[pce70211-bib-0074] Pammenter, N. W. , and C. Vander Willigen . 1998. “A Mathematical and Statistical Analysis of the Curves Illustrating Vulnerability of Xylem to Cavitation.” Tree Physiology 18: 589–593. 10.1093/treephys/18.8-9.589. 12651346

[pce70211-bib-0076] Peguero‐Pina, J. J. , D. Sancho‐Knapik , E. Barrón , J. J. Camarero , A. Vilagrosa , and E. Gil‐Pelegrín . 2014. “Morphological and Physiological Divergences Within *Quercus ilex* Support the Existence of Different Ecotypes Depending on Climatic Dryness.” Annals of Botany 114: 301–313. 24941998 10.1093/aob/mcu108 PMC4111378

[pce70211-bib-0077] Pellizzari, E. , J. J. Camarero , A. Gazol , G. Sangüesa‐Barreda , and M. Carrer . 2016. “Wood Anatomy and Carbon‐Isotope Discrimination Support Long‐Term Hydraulic Deterioration as a Major Cause of Drought‐Induced Dieback.” Global Change Biology 22: 2125–2137. 26790660 10.1111/gcb.13227

[pce70211-bib-0078] Petek‐Petrik, A. , P. Petrík , L. J. Lamarque , H. Cochard , R. Burlett , and S. Delzon . 2023. “Drought Survival in Conifer Species Is Related to the Time Required to Cross the Stomatal Safety Margin.” Journal of Experimental Botany 74: 6847–6859. 37681745 10.1093/jxb/erad352

[pce70211-bib-0080] Preisler, Y. , F. Tatarinov , J. M. Grünzweig , et al. 2019. “Mortality Versus Survival in Drought‐Affected Aleppo Pine Forest Depends on the Extent of Rock Cover and Soil Stoniness.” Functional Ecology 33: 901–912. 10.1111/1365-2435.13302.

[pce70211-bib-0081] R Core Team . 2024. R: A Language and Environment for Statistical Computing.

[pce70211-bib-0082] Riederer, M. and Müller, C. , ed. 2006. Biology of the Plant Cuticle. Blackwell. 10.1002/9780470988718.

[pce70211-bib-0083] Ripullone, F. , J. J. Camarero , M. Colangelo , and J. Voltas . 2020. “Variation in the Access to Deep Soil Water Pools Explains Tree‐to‐Tree Differences in Drought‐Triggered Dieback of Mediterranean Oaks.” Tree Physiology 40: 591–604. 32159804 10.1093/treephys/tpaa026

[pce70211-bib-0084] Rodriguez‐Dominguez, C. M. , A. Forner , S. Martorell , et al. 2022. “Leaf Water Potential Measurements Using the Pressure Chamber: Synthetic Testing of Assumptions Towards Best Practices for Precision and Accuracy.” Plant, Cell & Environment 45: 2037–2061. 10.1111/pce.14330. PMC9322401 35394651

[pce70211-bib-0085] Roskilly, B. , E. Keeling , S. Hood , A. Giuggiola , and A. Sala . 2019. “Conflicting Functional Effects of Xylem Pit Structure Relate to the Growth‐Longevity Trade‐Off in a Conifer Species.” Proceedings of the National Academy of Sciences 116: 15282–15287. 10.1073/pnas.1900734116. PMC6660759 31209057

[pce70211-bib-0086] Rowland, L. , A. C. L. Da Costa , D. R. Galbraith , et al. 2015. “Death From Drought in Tropical Forests Is Triggered by Hydraulics Not Carbon Starvation.” Nature 528: 119–122. 26595275 10.1038/nature15539

[pce70211-bib-0088] Schneider, C. A. , W. S. Rasband , and K. W. Eliceiri . 2012. “NIH Image to ImageJ: 25 Years of Image Analysis.” Nature Methods 9: 671–675. 10.1038/nmeth.2089. 22930834 PMC5554542

[pce70211-bib-0089] Sperry, J. S. , J. R. Donnelly , and M. T. Tyree . 1988. “A Method for Measuring Hydraulic Conductivity and Embolism in Xylem.” Plant, Cell & Environment 11: 35–40.

[pce70211-bib-0091] Torres‐Ruiz, J. M. , H. Cochard , M. Mencuccini , S. Delzon , and E. Badel . 2016. “Direct Observation and Modelling of Embolism Spread Between Xylem Conduits: A Case Study in Scots Pine.” Plant, Cell & Environment 39: 2774–2785. 10.1111/pce.12840. 27739597

[pce70211-bib-0090] Torres‐Ruiz, J. M. , H. Cochard , S. Delzon , et al. 2024. “Plant Hydraulics at the Heart of Plant, Crops and Ecosystem Functions in the Face of Climate Change.” New Phytologist 241: 984–999. 10.1111/nph.19463. 38098153

[pce70211-bib-0092] Torres‐Ruiz, J. M. , S. Jansen , B. Choat , et al. 2015. “Direct X‐Ray Microtomography Observation Confirms the Induction of Embolism Upon Xylem Cutting Under Tension.” Plant Physiology 167: 40–43. 25378693 10.1104/pp.114.249706 PMC4281005

[pce70211-bib-0093] Trugman, A. T. , L. D. L. Anderegg , W. R. L. Anderegg , A. J. Das , and N. L. Stephenson . 2001. “Why Is Tree Drought Mortality so Hard to Predict?” Trends in Ecology and Evolution (Personal Edition) 36: 520–532. 10.1016/j.tree.2021.02.001 33674131

[pce70211-bib-0094] Valeriano, C. , A. Gazol , M. Colangelo , and J. J. Camarero . 2021. “Drought Drives Growth and Mortality Rates in Three Pine Species Under Mediterranean Conditions.” Forests 12: 1700.

[pce70211-bib-0095] Valeriano, C. , A. Gazol , M. Colangelo , E. González de Andrés , and J. J. Camarero . 2021. “Modeling Climate Impacts on Tree Growth to Assess Tree Vulnerability to Drought During Forest Dieback.” Frontiers in Plant Science 12: 672855. 10.3389/fpls.2021.672855. 34512680 PMC8426521

[pce70211-bib-0097] Velasco‐Conde, T. , I. Yakovlev , J. P. Majada , I. Aranda , and Ø. Johnsen . 2012. “Dehydrins in Maritime Pine (*Pinus pinaster*) and Their Expression Related to Drought Stress Response.” Tree Genetics & Genomes 8: 957–973.

[pce70211-bib-0099] Voltas, J. , J. J. Camarero , D. Carulla , M. Aguilera , A. Ortiz , and J. P. Ferrio . 2013. “A Retrospective, Dual‐Isotope Approach Reveals Individual Predispositions to Winter‐Drought Induced Tree Dieback in the Southernmost Distribution Limit of Scots Pine.” Plant, Cell & Environment 36: 1435–1448. 10.1111/pce.12072 23346991

[pce70211-bib-0075] Williams, A. P. , C. D. Allen , A. K. Macalady , et al. 2013. “Temperature as a Potent Driver of Regional Forest Drought Stress and Tree Mortality.” Nature Climate Change 3: 292–297. 10.1038/nclimate1693.

