# Supplementary material for: Xylem Pit Anatomy and Minimum Leaf Conductance Drive Drought Mortality in Pinus pinaster
Source: Plant Cell Environ. 2025 Sep 30;49(1):279–94. doi: 10.1111/pce.70211 (PMC12675989; doi:10.1111/pce.70211)
Supplement: Supplementary file 1 — Figure S1: (a) Climate diagram and (b) monthly water budgets based on monthly climate data from the Daroca station (period 1920–2018). Figure S2: Seasonal climate data measured in the Daroca station. Lines indicate linear regression for variables showing significant (p < 0.05) trends according to Mann‐Kendall tests. Figure S3: Histogram showing historical data of annual climatic water balances (Daroca station, period 1920–2018) and the balance recorded in the severe 2017 drought (dotted vertical line). Figure S4: Daily climate data recorded during the monitoring period (2016–2020) when most measurements of wood anatomy, leaf variables, water potential and hydraulic conductivity were done. Plots show (a) mean maximum temperatures, (b) mean minimum temperatures, and (c) total precipitation. Data are from Daroca meteorological station and lines show mean values for annual cycles (period 1920–2020). Figure S5: Differences in the isotope (δ18O, δ2H) measurements made in water samples taken from xylem and soils during spring (23rd June) and late summer (8th September) in 2020. Xylem samples were obtained from declining (downward black triangles) and non‐declining P. pinaster (upward white triangles) and from Q. ilex trees (green circles). Soil samples were taken at 0–15 cm (light grey squares) and 15–30 cm (dark grey squares) depths. Figure S6: Relationships between water potential and specific hydraulic conductivity (Ks) in declining (orange symbols and lines) and non‐declining (blue symbols and lines) trees. Sampling date: June 2020. Figure S7: Relationships between water potential and percent loss of conductivity (PLC) in declining (orange symbols and lines) and non‐declining (blue symbols and lines) trees. The horizontal dashed line shows the water potentials at which 50 % of hydraulic conductivity is lost (P50). Sampling date: June 2020. The P50 value of declining trees (3.18 MPa) was significantly (p < 0.05) lower than that measured in non‐declining trees (3.34 MPa) [file PCE-49-279-s001.docx]

**Supporting Information**

| Vigor class or tree species | 23rd June | | 8th September | |
| --- | --- | --- | --- | --- |
|  | δ^18^O (‰) | δ^2^H (‰) | δ^18^O (‰) | δ^2^H (‰) |
| Declining trees | -6.53 ± 0.10a | -51.64 ± 0.45a | -3.45 ± 0.25a | -43.26 ± 0.82a |
| Non-declining trees | -6.42 ± 0.12a | -54.39 ± 0.49b | -2.81 ± 0.23a | -47.05 ± 0.89b |
| *Quercus ilex* | -8.23 ± 0.20b | -66.70 ± 1.03c | -10.07 ± 0.39b | -79.17 ± 1.75c |
| *F* | 33.01 | 88.55 | 131.08 | 171.09 |
| *p* | < 0.001 | < 0.001 | < 0.001 | < 0.001 |

**Table S1.** Data on xylem and soil water isotopes measured in late June and early September 2020. Values are means ± SE. The last lines show *F* statistic corresponding to one-way ANOVAs between the three groups of trees, whereas different letters indicate significant (*p* < 0.05) differences between groups (*t* tests).

| Vigor class (code) | Starch (%) | Soluble sugars (%) | Total NSCs (%) |
| --- | --- | --- | --- |
| Declining trees (D) | 1.78 ± 0.09 | 1.05 ± 0.08 | 2.83 ± 0.08 |
| Non-declining trees (ND) | 1.93 ± 0.13 | 1.14 ± 0.10 | 3.07 ± 0.19 |
| *t* | -0.94 | -0.69 | -1.15 |
| *p* | 0.36 | 0.49 | 0.26 |

**Table S2.** Concentrations of NSCs measured in sapwood samples. Values are means ± SE. Different letters indicate significant (*p* < 0.05) differences between groups of trees (*t* tests).

| (a) |
| --- |
| 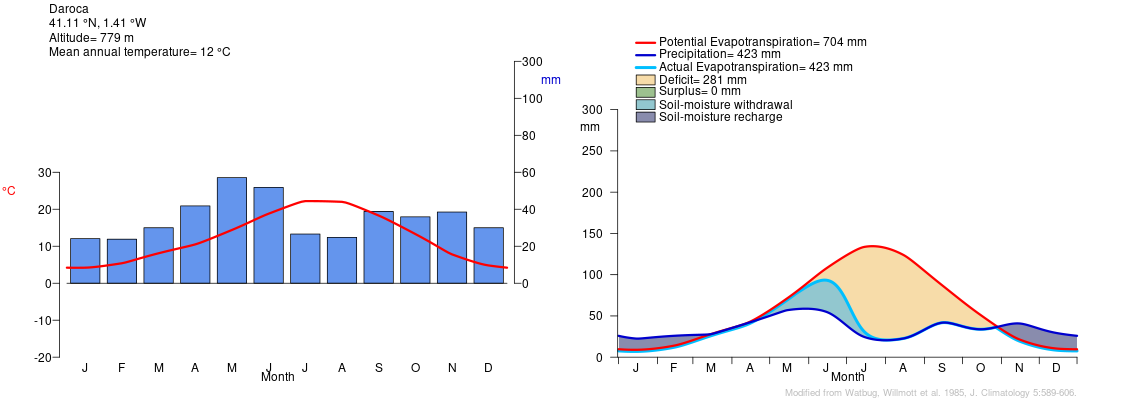 |
| (b) |
| 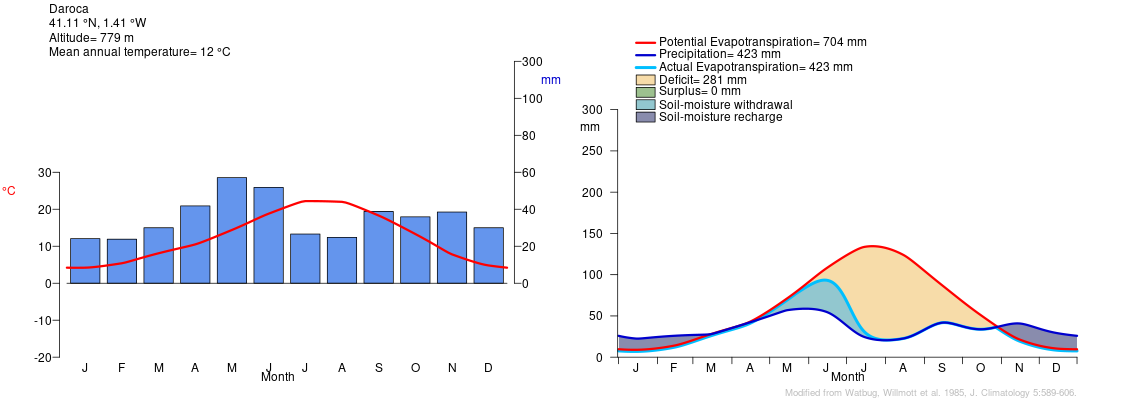 |

**Figure S1.** (a) Climate diagram and (b) monthly water budgets based on monthly climate data from the Daroca station (period 1920−2018).

**Figure S2.** Seasonal climate data measured in the Daroca station. Lines indicate linear regression for variables showing significant (*p* < 0.05) trends according to Mann-Kendall tests.

**Figure S3.** Histogram showing historical data of annual climatic water balances (Daroca station, period 1920−2018) and the balance recorded in the severe 2017 drought (dotted vertical line).

**Figure S4.** Daily climate data recorded during the monitoring period (2016−2020) when most measurements of wood anatomy, leaf variables, water potential and hydraulic conductivity were done. Plots show (a) mean maximum temperatures, (b) mean minimum temperatures, and (c) total precipitation. Data are from Daroca meteorological station and lines show mean values for annual cycles (period 1920−2020).

**Figure S5.** Differences in the isotope (δ^18^O, δ^2^H) measurements made in water samples taken from xylem and soils during spring (23^rd^ June) and late summer (8^th^ September) in 2020. Xylem samples were obtained from declining (downward black triangles) and non-declining *P. pinaster* (upward white triangles) and from *Q. ilex* trees (green circles). Soil samples were taken at 0-15 cm (light grey squares) and 15-30 cm (dark grey squares) depths.

***
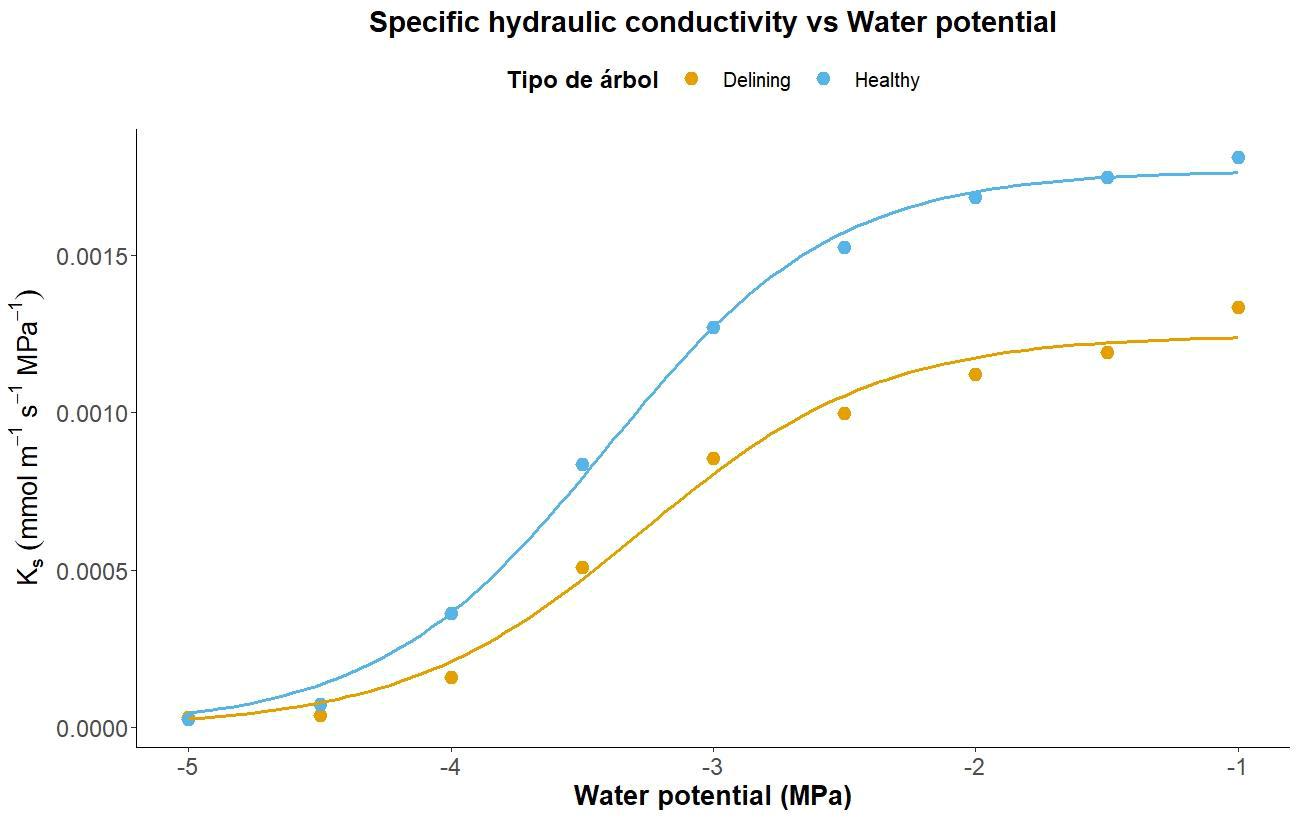
***

**Figure S6.** Relationships between water potential and specific hydraulic conductivity (K_s_) in declining (orange symbols and lines) and non-declining (blue symbols and lines) trees. Sampling date: June 2020.

*
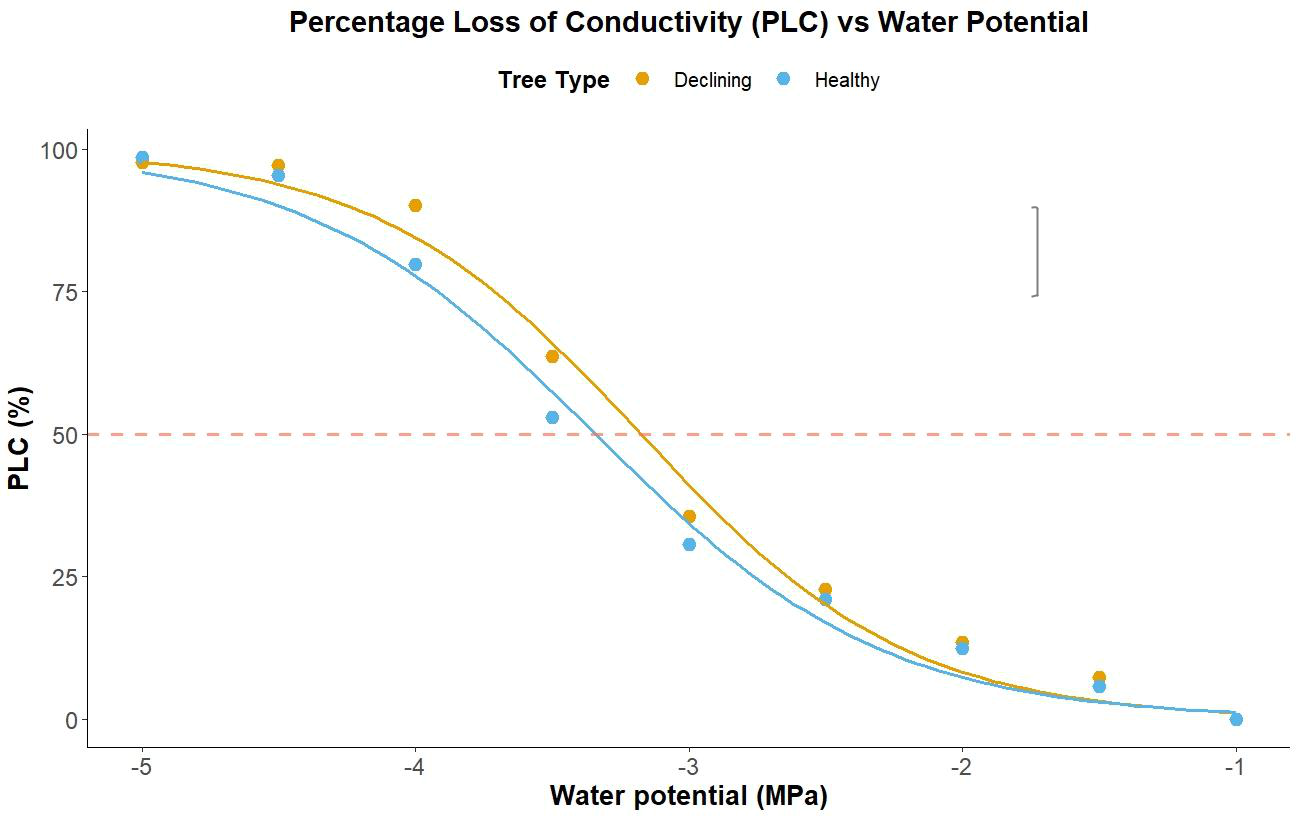
*

**Figure S7.** Relationships between water potential and percent loss of conductivity (PLC) in declining (orange symbols and lines) and non-declining (blue symbols and lines) trees. The horizontal dashed line shows the water potentials at which 50 % of hydraulic conductivity is lost (P50). Sampling date: June 2020. The P50 value of declining trees (3.18 MPa) was significantly (*p* < 0.05) lower than that measured in non-declining trees (3.34 MPa).

**Figure S8.** Significant negative correlations (Pearson coefficients) found between mean (a) crown defoliation or (b) *g_min_* and late-July midday water potential. Variables were measured in 2020.
